# Divergent Host-Microbe Interaction and Pathogenesis Proteins Detected in Recently Identified *Liberibacter* Species

**DOI:** 10.1128/spectrum.02091-22

**Published:** 2022-07-28

**Authors:** Allison K. Hansen, Ariana N. Sanchez, Younghwan Kwak

**Affiliations:** a Department of Entomology, University of California, Riverside, California, USA; University of Nebraska - Lincoln

**Keywords:** *Candidatus* (*Ca.*) Liberibacter, *Ca.* Liberibacter capsica, purifying selection, Tad pilus complex, horizontal gene transfer

## Abstract

*Candidatus* (*Ca.*) Liberibacter taxa are economically important bacterial plant pathogens that are not culturable; however, genome-enabled insights can help us develop a deeper understanding of their host-microbe interactions and evolution. The draft genome of a recently identified *Liberibacter* taxa, *Ca.* Liberibacter capsica, was curated and annotated here with a total draft genome size of 1.1 MB with 1,036 proteins, which is comparable to other *Liberibacter* species with complete genomes. A total of 459 orthologous clusters were identified among *Ca.* L. capsica, *Ca.* L. asiaticus, *Ca.* L. psyllaurous*, Ca.* L. americanus, *Ca.* L. africanus, and *L. crescens*, and these genes within these clusters consisted of housekeeping and environmental response functions. We estimated the rates of molecular evolution for each of the 443 one-to-one ortholog clusters and found that all *Ca.* L. capsica orthologous pairs were under purifying selection when the synonymous substitutions per synonymous site (dS) were not saturated. These results suggest that these genes are largely maintaining their conserved functions. We also identified the most divergent single-copy orthologous proteins in *Ca.* L. capsica by analyzing the ortholog pairs that represented the highest nonsynonymous substitutions per nonsynonymous site (dN) values for each pairwise comparison. From these analyses, we found that 21 proteins which are known to be involved in pathogenesis and host-microbe interactions, including the Tad pilus complex, were consistently divergent between *Ca.* L. capsica and the majority of other *Liberibacter* species. These results further our understanding of the evolutionary genetics of *Ca*. L. capsica and, more broadly, the evolution of *Liberibacter*.

**IMPORTANCE** *“Candidatus”* (*Ca.*) Liberibacter taxa are economically important plant pathogens vectored by insects; however, these host-dependent bacterial taxa are extremely difficult to study because they are unculturable. Recently, we identified a new *Ca*. Liberibacter lineage (*Ca.* Liberibacter capsica) from a rare insect metagenomic sample. In this current study, we report that the draft genome of *Ca.* Liberibacter capsica is similar in genome size and protein content compared to the other *Ca.* Liberibacter taxa. We provide evidence that many of their shared genes, which encode housekeeping and environmental response functions, are evolving under purifying selection, suggesting that these genes are maintaining similar functions. Our study also identifies 21 proteins that are rapidly evolving amino acid changes in *Ca.* Liberibacter capsica compared to the majority of other *Liberibacter* taxa. Many of these proteins represent key genes involved in *Liberibacter*-host interactions and pathogenesis and are valuable candidate genes for future studies.

## INTRODUCTION

Microbiome sequencing during the last few decades has revealed two critical observations. First is that the overwhelming majority of microbes have never been cultured in a laboratory, and second is that this “unseen majority” have important ecological and economic impacts (1). For these fastidious microbes, genome-enabled technologies facilitate a deeper understanding of gene function, host-microbe interactions, and evolution (2). One group of microbes that are unculturable but extremely important to identify and understand at an evolutionary genetic level are the economically important group of plant pathogens that belong to the bacterial genus *Liberibacter* (3, 4). These bacteria are harbored and exclusively vectored by sap-sucking insects in the superfamily Psylloidea (5). In consequence, uncovering *Liberibacter* genes that are important for both insect and plant survival is important for elucidating how *Liberibacter* responds and interacts in both of its hosts’ environments (6).

A total of nine *Liberibacter* species have been identified to date, and these include *Candidatus* (*Ca.*) Liberibacter capsica, *Ca*. L. psyllaurous, *Ca*. L. asiaticus, *Ca*. L. africanus, *Ca*. L. americanus, *Ca*. L. europaeus, *Ca*. L. brunswickensis, *Ca*. L. ctenarytainae, and *L*. *crescens* (5). Only one culturable *Liberibacter* species, *L. crescens*, has been identified and sequenced to date, and it is the most divergent species within the *Liberibacter* genus (7). The *Liberibacter* taxa *Ca*. L. africanus, *Ca*. L. americanus, and especially *Ca*. L. asiaticus are all associated with the devastating disease of citrus called Huanglongbing (also known as citrus greening disease) (8), and *Ca*. L. psyllaurous widely infects plants within the families Solanaceae (9), Apiaceae (10), and Urticaceae (11) and is associated with psyllid yellows (9) and zebra chip disease (12) in the economically important crop of potato. In addition to not being culturable, *Candidatus* Liberibacter species are hard to sequence with high coverage because these fastidious microbes need to be sequenced in either their psyllid or plant host tissues (13). Nevertheless, four unculturable *Liberibacter* species have been fully sequenced to date: *Ca.* L. asiaticus, *Ca.* L. psyllaurous, *Ca.* L. americanus, *Ca.* L. africanus (14).

The most recent *Ca.* Liberibacter species identified using a metagenomic and phylogenetic approach is *Ca.* Liberibacter capsica (5). This metagenomic sample was sequenced and identified from a rare DNA sample extracted from a single pair of adult male and female psyllids that belonged to the psyllid species *Russelliana capsici*. These psyllids were collected from Brazil for use in a 16S rRNA analysis and were unexpectedly infected with the new *Ca.* Liberibacter taxa, *Ca.* L. capsica (5). The psyllid *R. capsici* is a pest on pepper (*Capsicum annuum*) in Argentina and Brazil (15); however, it is unknown if *Ca.* Liberibacter capsica can infect the psyllid’s host plant and/or cause plant disease at this time.

To further understand *Ca.* Liberibacter capsica’s biology and evolution through a genome-enabled approach, we curated and annotated the draft genome assembly of *Ca.* Liberibacter capsica to determine how this new taxon is similar to and different from other fully sequenced *Liberibacter* genomes, such as *L. crescens*, *Ca.* L. asiaticus, *Ca.* L. psyllaurous, *Ca.* L. americanus, and *Ca.* L. africanus. We also identified orthologous gene clusters in *Ca.* Liberibacter capsica and determined which one-to-one orthologs are evolving due to natural selection. We further identified orthologous proteins in *Ca.* Liberibacter capsica that display the highest rates of nonsynonymous substitutions per nonsynonymous sites (dN) compared to other *Liberibacter* taxa. Based on these evolutionary analyses, we highlight candidate proteins in *Ca.* Liberibacter capsica that may be important for host-microbe interactions in the evolution of this *Liberibacter* lineage.

## RESULTS

### Annotation of *Ca.* Liberibacter capsica’s draft genome.

The draft genome of *Ca.* Liberibacter capsica (5) is similar in total nt length, 1,111,229 nt (1,626 total contigs), compared to other fully sequenced reference *Liberibacter* species that are fastidious and nonculturable (Table 1). A total of 1,036 proteins were annotated here from the draft genome of *Ca.* Liberibacter capsica (5), which is comparable to the average number of genes (~1,122) annotated in other *Liberibacter* species with complete genomes (Table 1). Nevertheless, 40% of the 736 single copy orthologs which are present in the five reference *Liberibacter* species genomes, based on OrthoVenn2, are missing from the draft genome of *Ca.* Liberibacter capsica. These missing single copy orthologs are primarily comprised of genes involved in genetic information processing, indicating that the draft genome from this rare and limited metagenomic sample is not fully complete (Table S1). In consequence, the evolutionary analyses presented here are conservative and are based only on *Liberibacter* homologs that are present in the *Ca.* Liberibacter capsica draft genome.

**TABLE 1:** Genome statistics of the draft genome of *Ca*. Liberibacter capsica compared to fully sequenced reference genomes of *Liberibacter* species

Species	NCBI iD	nt length	Number of proteins	Clusters	Singletons
*Ca*. Liberibacter capsica	This study	1,111,229	1,036	580	409
*Ca*. Liberibacter africanus	NZ_CP004021.1	1,192,232	1,075	912	131
*Ca*. Liberibacter americanus	NC_022793.1	1,195,201	992	897	69
*Ca*. Liberibacter asiaticus	NZ_CP019958.1	1,225,162	1,082	916	131
*Ca*. Liberibacter psyllaurous	NC_014774.1	1,258,278	1,132	955	95
Liberibacter crescens	NZ_CP010522.1	1,522,119	1,331	871	396

### Identification of orthologous gene clusters in *Ca.* Liberibacter capsica.

**FIG 1:**
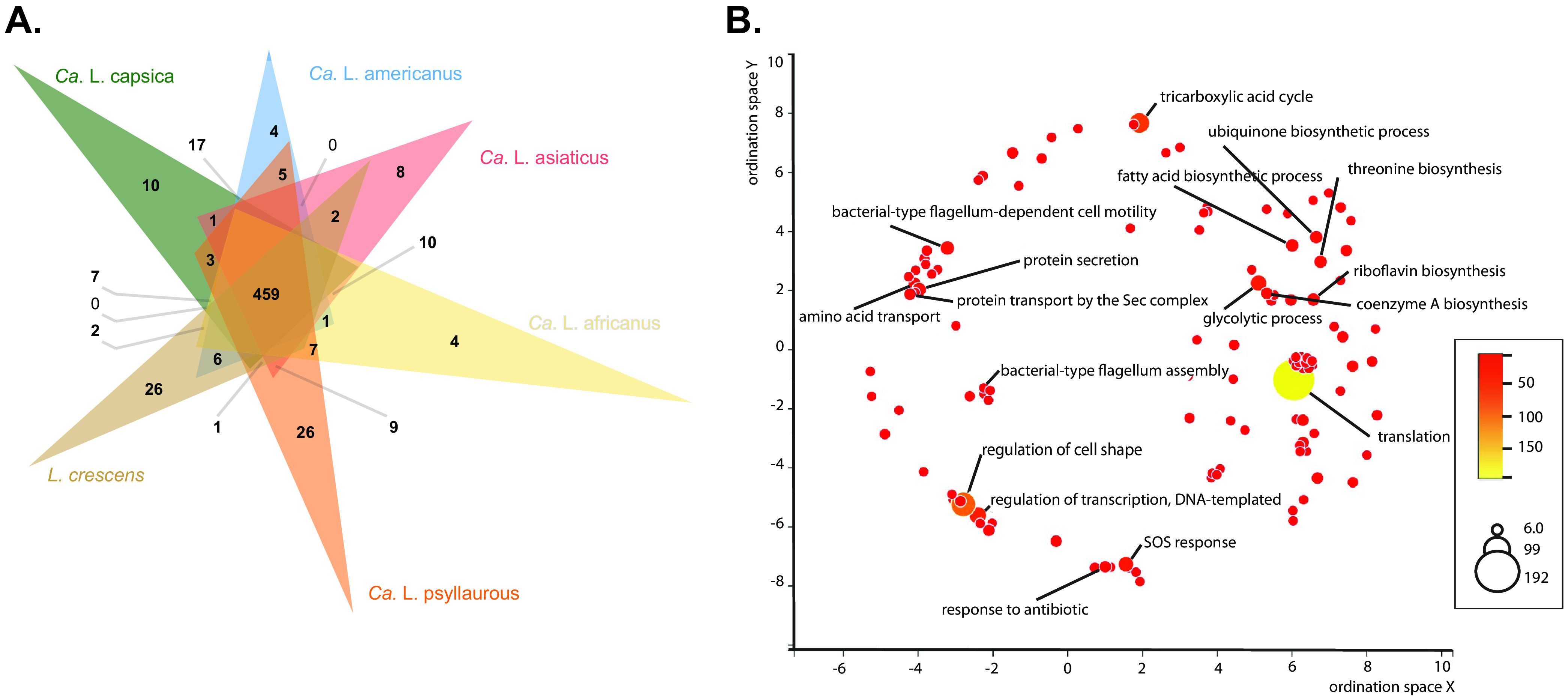
Orthologous gene clusters among *Ca.* L. capsica, *Ca.* L. asiaticus, *Ca.* L. psyllaurous, *Ca.* L. americanus, *Ca.* L. africanus, and *L. crescens.* (A) Overlap of orthologous gene clusters that are unique and shared between the six *Liberibacter* species. Numbers on the Venn diagram indicate the number of orthologous clusters shared by the species with overlapping sectors. Note that multiple genes per taxa can be present within an orthologous gene cluster. See Tables S2 and S3 for detail. (B) A total of 459 orthologous clusters are shared among all six Liberibacter taxa, and these clusters belong to 172 unique biological GO term categories, which are represented as circles in the figure (see Table S2 for detail). Multidimensional scaling (MDS) was used in Revigo to reduce the dimensionality of the matrix for these 172 GO terms based on pairwise similarities in biological function. The size of the circle and color indicates the relative number of proteins within each unique GO term category. The top 17 gene clusters that contain the most proteins are labeled in the figure.

**FIG 2:**
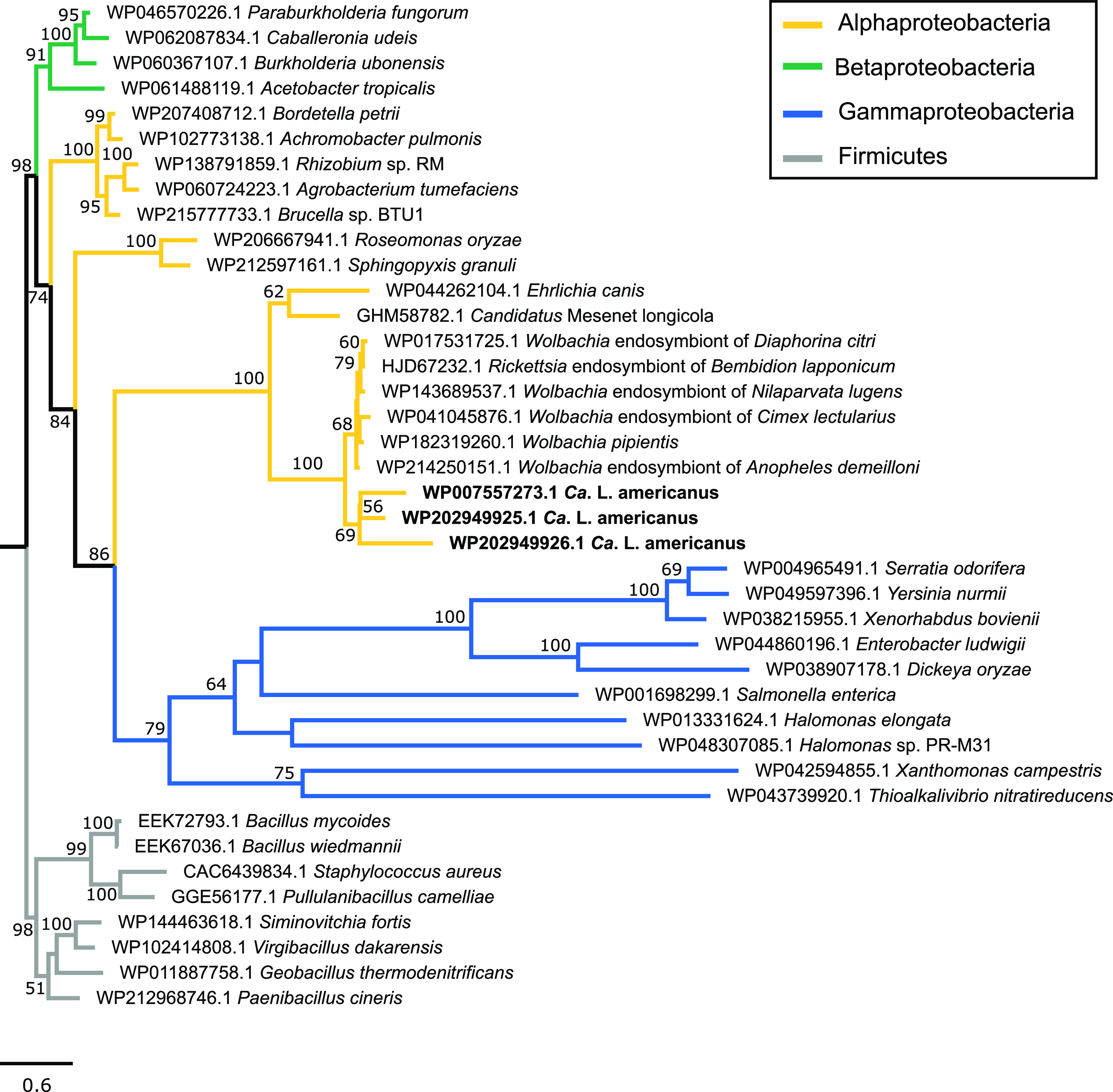
Phylogenetic analysis of MFS transporter proteins in *Ca*. L. americanus. The tree was rooted with the Firmicute outgroup. Bootstrap values are indicated for nodes with 50% or above.

A total of 459 orthologous clusters containing 2,791 genes (Table S2), which consist of 443 single copy orthologs (Table S3), are shared among *Ca.* L. capsica, *Ca.* L. asiaticus, *Ca.* L. psyllaurous, *Ca.* L. americanus, *Ca.* L. africanus, and *L. crescens* (Fig. 1A). Approximately 91.2% of these gene clusters belong to GO term categories (Table S2). The GO term categories that contain the most proteins include those that are involved in housekeeping functions, stress response, pathogenesis, and host-microbe interactions (Table S2; Fig. 1B). A total of 10, 8, 26, 4, 8, and 26 unique orthologous gene clusters were found in *Ca.* L. capsica, *Ca.* L. asiaticus, *Ca.* L. psyllaurous, *Ca.* L. americanus, *Ca.* L. africanus, and *L. crescens*, respectively (Fig. 1A). The 10 unique orthologous clusters in *Ca.* L. capsica were composed of 51 genes that have no annotations based on Swiss-Prot, GO Terms, or the best BLAST hit based on the NCBI nr database. Two of these clusters contained the majority of these genes (14 and 10 genes), and they were relatively short in amino acid length and annotated as hypothetical. Some of these hypothetical proteins occurred on contigs alone or with other *Liberibacter* orthologous proteins. The majority of novel orthologous clusters found in other *Liberibacter* species also were not associated with annotations based on Swiss-Prot, GO Terms, or the best BLAST hit based on the NCBI nr database. However, there were several cluster(s) identified in *Ca.* L. americanus (N = 1), *Ca.* L. asiaticus (N = 2), *Ca.* L. psyllaurous (N = 1), and *L. crescens* (N = 9) for which we could provide functional assignments (Table 2). Liberibacter crescens annotations were primarily associated with transporters, cell membrane components, and pathogenicity related factors (Table 2). In *Ca.* L. americanus, one cluster that consisted of three proteins that belong to the Major Facilitator Superfamily (MFS) based on Swiss-Prot (P16482) and NCBI BLASTP were annotated by Prokka as two proline/betaine (ProP) transporters and one glycine betaine/proline/ectoine/pipecolic acid transporter (OusA) (Table 2). Interestingly, based on our phylogenetic analysis, all three of these genetically distinct MFS transporters appear to be related to a *Wolbachia* ancestor, potentially suggesting that there was a horizontal gene transfer event from *Wolbachia* into the genome of *Ca.* L. americanus (Fig. 2).

**TABLE 2:** Annotated protein clusters that are unique for each *Liberibacter* species

Species	Protein count	Swiss-Prot hit	GO annotation
*Ca*. Liberibacter americanus	3	P16482	GO:0006101; P:citrate metabolic process
*Ca*. Liberibacter asiaticus	3	Q9PL92	GO:0006260; P:DNA replication
	2	P10484	GO:0009307; P:DNA restriction-modification system
*Ca*. Liberibacter psyllaurous	3	P78285	GO:0009253; P:peptidoglycan catabolic process
	2	P44189	NA
Liberibacter crescens	6	Q44664	GO:0016021; C:integral component of membrane
	4	Q9T1T5	GO:0009253; P:peptidoglycan catabolic process
	2	Q6GJ96	GO:0015293; F:symporter activity
	2	P0A535	GO:0006535; P:cysteine biosynthetic process from serine
	2	P31224	GO:0042493; P:response to drug
	2	P23214	GO:0016747; F:transferase activity
	2	B9JXW8	GO:0000105; P:histidine biosynthetic process
	2	P45082	GO:0042883; P:cysteine transport
	2	Q8G3D4	GO:0006865; P:amino acid transport

A total of 409 proteins from *Ca.* L. capsica were singletons (Table 1) and did not form significant orthologous clusters with proteins from other Liberibacter species or itself. Only 48 of these singletons had significant hits to the NCBI nr database, and most of them (16) had a best hit to hypothetical proteins, phage tail proteins, and integrases in other *Liberibacter* species; however, they were not clustered as significant orthologous clusters by OrthoVenn2. The remaining three singletons had 60 to 90% sequence similarity to hypothetical proteins that belong to other Alphaproteobacterial species.

### Identification of genes experiencing natural selection in *Ca.* Liberibacter capsica.

We analyzed the single copy orthologous genes (N = 443) that were found in all six *Liberibacter* species for signatures of natural selection by calculating the dN (nonsynonymous substitutions per nonsynonymous site) and dS (synonymous substitutions per synonymous site) values among each species pair with our target species, *Ca.* L. capsica. For all 1:1 single copy orthologous pairs in which dS was not saturated, the dN/dS ratio was <1, indicating that purifying selection is widespread among *Liberibacter* single copy gene orthologs. For the species pair *Ca.* L. capsica and *Ca.* L. americanus, we found 439 gene pairs with an average dN/dS ratio of 0.13 (stdev = 0.08). A total of 365 gene pairs are under purifying selection between *Ca.* L. capsica and *Ca.* L. psyllaurous and had an average dN/dS ratio of 0.09 (stdev = 0.04). For the species pair *Ca.* L. capsica and *Ca.* L. asiaticus, we found 315 gene pairs with an average dN/dS ratio of 0.09 (stdev = 0.06), and for the species pair *Ca.* L. capsica and *Ca.* L. africanus, we found 285 gene pairs with an average dN/dS ratio of 0.09 (stdev = 0.04). For the most divergent species pair, *Ca.* L. capsica and *Ca.* L. crescens, although we found only 38 gene pairs that did not have saturated dS values, the average dN/dS ratio was still just 0.12 (stdev = 0.09). As expected, based on Swiss-Prot and GO term annotations, all gene pairs under purifying selection primarily represent housekeeping genes (Table S3).

**FIG 3:**
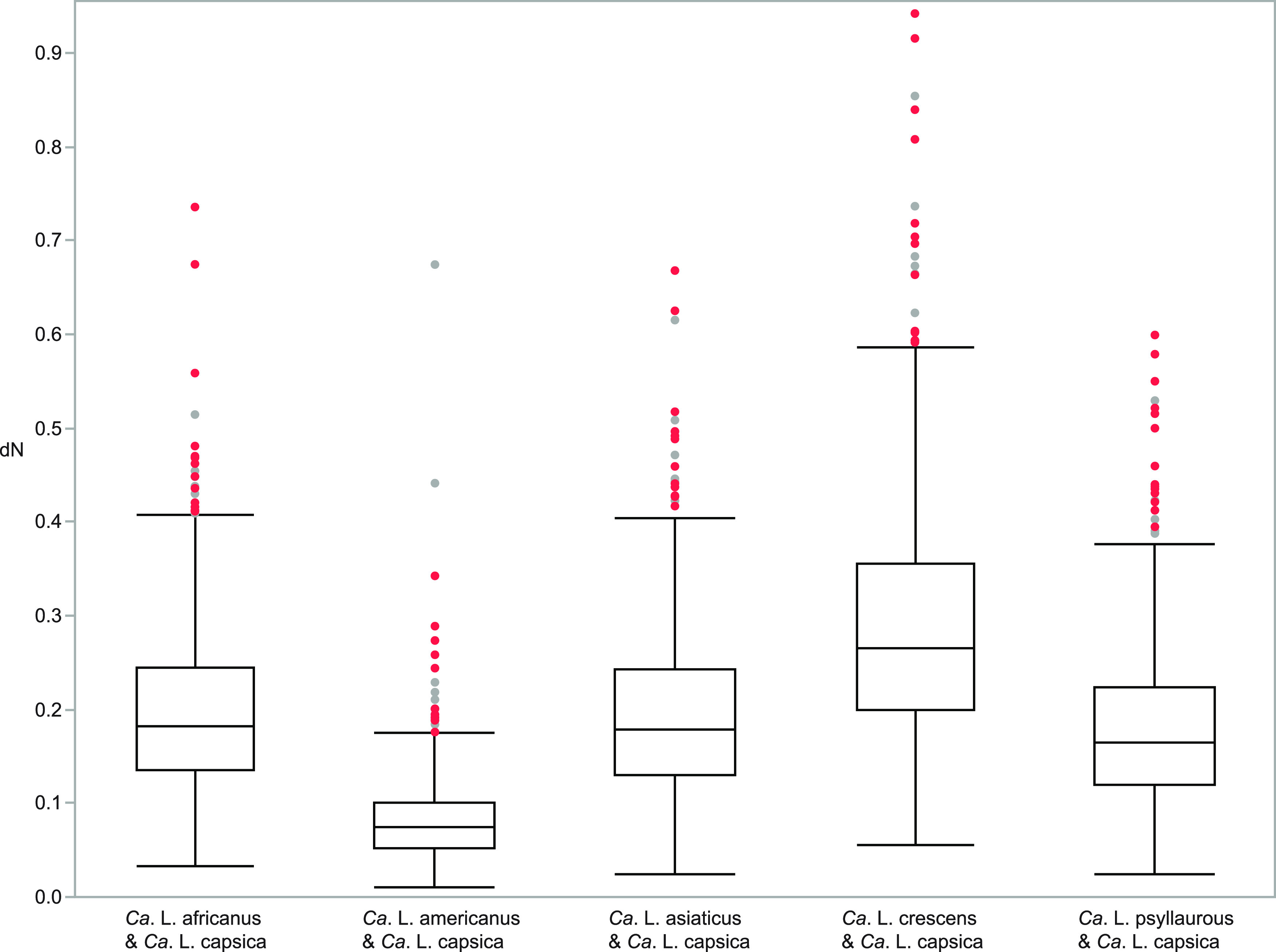
Box and whisker plots showing the divergence between 1:1 orthologs of *Ca.* L. capsica and other *Liberibacter* species based on nonsynonymous substitutions per nonsynonymous site (dN). All points shown are outliers, and red points are dN values for proteins that are consistently in the top 10% per species pair for all Liberibacter species comparisons. All dN values are shown per pairwise comparison (N = 443).

**FIG 4:**
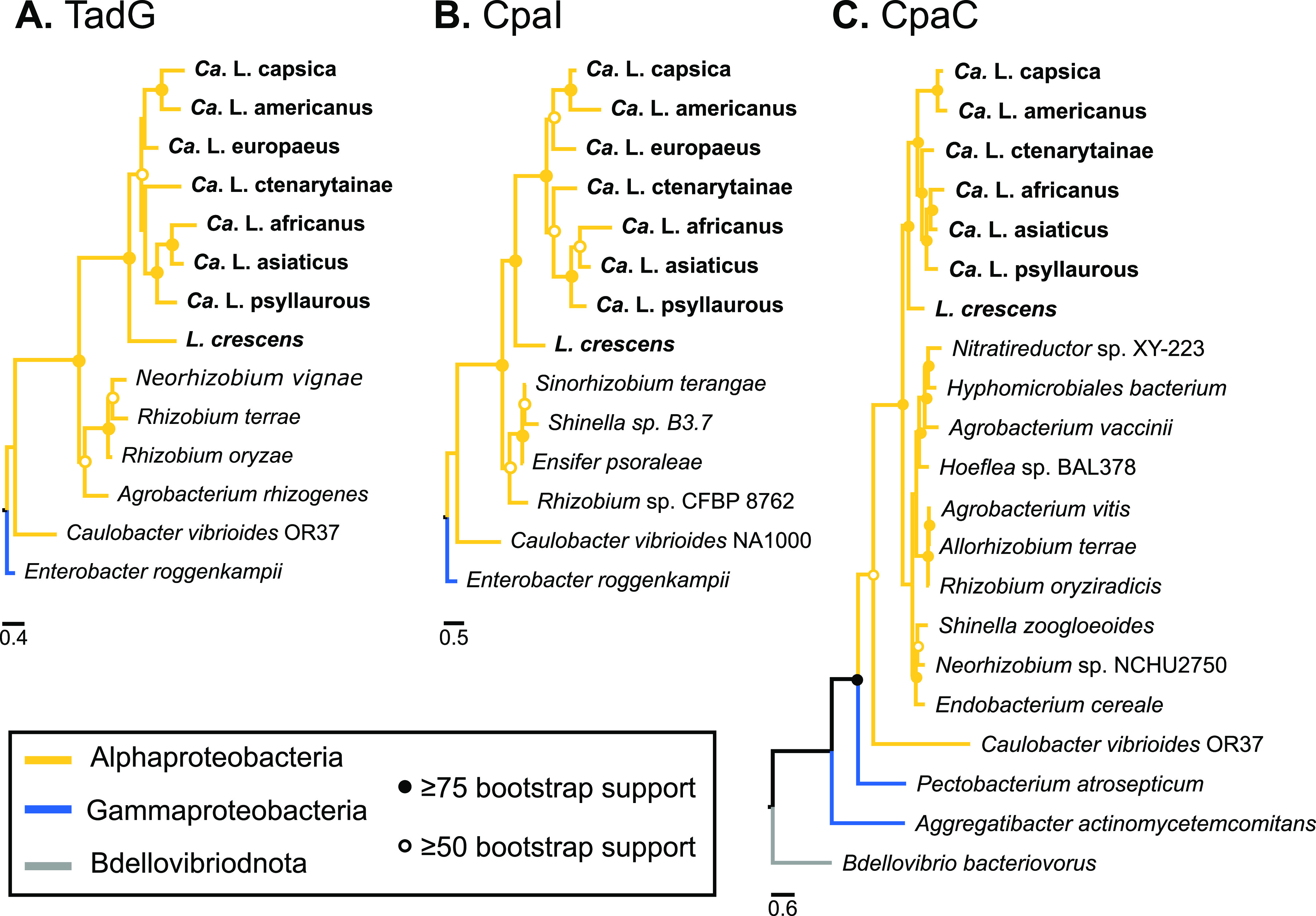
Phylogenetic analyses of Tad pilus genes in *Liberibacter* taxa and relatives for (A) TadG, (B) CpaI, and (C) CpaC genes. Branches are colored according to their bacterial classes within the Proteobacteria, and ranges of bootstrap values are indicated by filled or open circles as indicated in the key. See Fig. S1, S2, and S3 in the supplemental material for trees with detailed taxa names and accession numbers.

We also identified the most divergent single copy orthologous proteins in *Ca.* L. capsica by analyzing ortholog pairs that represented the highest dN values (top 10%) for each pairwise comparison (N = 44) (Fig. 3). Within the top 10% of dN values for each pairwise comparison, the same 16 proteins were consistently found between *Ca.* L. capsica and the other five *Liberibacter* species (Table 3). When comparing *Ca.* L. capsica between all four unculturable *Ca.* Liberibacter species, 21 proteins were consistently found in the top 10% of dN (Table 3; Fig. S1). Seven of these latter proteins may be important for host-microbe interactions, such as the three Tad pilus apparatus proteins, CpaC, TadG/RcpC, and CpaI (Table 3). The Tad pilus complex is conserved in all *Liberibacter* species and is involved in host cell interactions in addition to the uptake of DNA (17, 18). Phylogenetic analyses for all three rapidly evolving Tad pilus proteins show significant clustering of all *Liberibacter* taxa, with branch supports between 81 and 100%, and all share a most recent common ancestor with taxa in the Alphaproteobacteria (Fig. 4; Fig. S1 to S3). Upon further investigation of Tad pilus genes, we identified a total of 9 out 13 Tad pilus genes in *Ca.* L. capsica’s draft genome using BLASTP: *cpaC*, *cpaD*, *cpaE*, *cpaF*, *cpaI*, *tadB*, *tadC*, *tadD*, and *tadG* (Table S4). Out of these Tad pilus proteins, CpaD was also in the top 10% of dN values for all *Ca*. L. capsica pairwise comparisons, except for that with its nearest relative, Ca. L. americanus (Table S4).

**TABLE 3:** Annotations of the most diverged one-to-one orthologs between *Ca.* L. capsica and other Liberibacter species

Protein description^*a*^	Scientific name	% identity	E value	KO^*b*^
4-hydroxybenzoate octaprenyltransferase	Ca. L. europaeus	73	8.00E-70	K03179
1 pyruvate dehydrogenase complex dihydrolipoamide acetyltransferase	Ca. L. americanus	69.9	2.55E-72	K00627
trypsin-like peptidase domain-containing protein, DegP*	Ca. L. americanus	64.1	6.30E-216	K04771
hypothetical protein*	Ca. L. americanus	68.1	1.18E-72	
DNA polymerase III subunit chi*	Ca. L. europaeus	67.9	3.05E-19	
PAS domain S-box protein*	Ca. L. americanus	72.9	0	
hypothetical protein, COG5462*	Ca. L. americanus	65.5	7.63E-28	
Flp pilus assembly protein, secretin CpaC	Ca. L. americanus	74.8	6.17E-208	K02280
Ribosomal protein L10*	Ca. L. americanus	75	2.66E-47	K02864
preprotein translocase subunit SecG*	Ca. L. americanus	60.3	1.51E-37	K03075
hypothetical protein*	Ca. L. americanus	70.8	7.00E-236	
pilus assembly protein, TadG/RcpC*	Ca. L. americanus	51.6	7.22E-23	
DUF1217 domain-containing protein; similar to FlgF*	Ca. L. americanus	68.3	5.47E-145	
50S ribosomal protein L9	Ca. L. americanus	65.7	4.80E-20	K02939
pilus assembly protein, N-terminal domain-containing, CpaI*	Ca. L. americanus	44.8	4.58E-36	
hypothetical protein*	Ca. L. americanus	70.5	2.66E-85	K11719
outer membrane protein assembly factor BamA*	Ca. L. americanus	70.6	2.69E-306	K07277
RIP metalloprotease RseP*	Ca. L. americanus	62.4	2.18E-118	K11749
M23 family metallopeptidase	Ca. L. americanus	61.9	6.15E-109	
disulfide bond formation protein B*	Ca. L. americanus	73.7	8.12E-78	
peptidylprolyl isomerase*	Ca. L. americanus	69.3	1.56E-89	K03771

a Proteins that are consistently the most divergent (top 10% of dN values) for all *Liberibacter* pairwise comparisons with *Ca.* L. capsica are indicated with an asterisk (*). Proteins that are not marked with an asterisk are consistently the highest values of dN (top 10%) for all Liberibacter pairwise comparisons except for the most divergent species, *L. crescens*. Protein descriptions, closest sequence match, identity, and E values are based on BLASTP against the NCBI nr database.

b KO term based on BlastKOALA‘s best hit.

The remaining four proteins that may be involved in host-microbe interactions and were consistently found in the top 10% of dN values are SecG, DegP, RseP, and a M23 family metallopeptidase protein (Table 3; Fig. S4). SecG is associated with the SecYEG complex, which is a Sec-dependent secretory pathway that is important for translocating virulence factors in intracellular pathogens (19). The two proteins DegP and RseP are known to protect bacterial pathogens from temperature and other stressors in the host environment (20, 21). The DegP protein was identified with significant domain hits to the periplasmic serine protease and PDZ domains, and the RseP protein was identified with significant domain hits to the peptidase M50 and PDZ domains based on significant NCBI conserved domain hits (Table 3). The M23 family metallopeptidase protein was identified with significant domain hits to the NlpD domain and to the M23 peptidase superfamily based on significant NCBI conserved domain hits (Table 3). This M23 family metallopeptidase protein is known to be important for lysing bacterial cell wall peptidoglycan for strain competition and for peptidoglycan turnover, and it was also identified as a virulence factor for Pseudomonas aeruginosa (22).

## DISCUSSION

Here, we identify patterns of genome-wide sequence evolution in *Ca*. L. capsica, including a group of proteins that are diverging rapidly (Table 3; Fig. S4). Several of these proteins are known to be important for host-microbe interactions and pathogenesis in *Liberibacter* (6, 17, 18, 23) and therefore have potential to further inform us on the evolution of *Liberibacter*-host interactions. Moreover, protein changes in these candidate genes may have major implications for the lineage-specific adaptation of *Ca*. L. capsica to its host environment(s). Currently, *Russelliana capsici* is the only known psyllid host of *Ca*. L. capsica. The psyllid *R. capsica* has been observed to feed on *Capsicum annuum* across its native distribution in Brazil (Minas Gerais, Paraná, Santa Catarina, São Paulo) and Argentina (Buenos Aires, Entre Ríos) (24). As such, it will be of interest for future studies to determine if this new *Ca*. Liberibacter taxa can infect plant tissue as well and potentially be a plant pathogen and/or commensal on the psyllid’s host plant (5).

Interestingly, four proteins (CpaC, CpaD, CpaI, and TadG) that work together to form the Tad pilus complex are rapidly diverging in *Ca*. L. capsica’s genome compared to the majority of *Liberibacter* taxa (Table S4). The Tad pilus complex is hypothesized to be important in the attachment and colonization of psyllid midguts because the Tad pilus genes are conserved in all *Liberibacter* taxa, and these Tad pilus genes are expressed more highly in psyllids compared to plants (17). Recent findings in Cai et al. (18) suggest that the Tad pilus complex may also be important in DNA uptake in both psyllid and plant host environments due to the demonstration of twitching motility in *Liberibacter* and the fact that *Liberibacter* taxa have lost the machinery to make their own nucleotides. Here, two Tad pilus associated proteins that we identified to be highly diverging in *Ca*. L. capsica are associated with the outer membrane of the Tad pilus complex (Table S4). Specifically, CpaC is the outer membrane secretin complex, and CpaD is linked to CpaC on the outer membrane and helps assemble CpaC (17, 25). As for the remaining two Tad pilus proteins that are diverging in *Ca*. L. capsica, the TadG protein may serve as an anchor for the Flp pilus, and CpaI is hypothesized to be important in the Tad pilus assembly in *Liberibacter* (17). It will be of interest for future studies to determine if these amino acid changes have changed the function, binding specificity, and/or role of the Tad pilus complex in *Ca*. L. capsica compared to other *Liberibacter* taxa.

Other proteins that are highly diverged in *Ca.* L. capsica, such as DegP and RseP, are important for a microbe’s adaptation to the host environment because of their role in stress response. For example, DegP is a periplasmic serine protease that is essential for survival in high temperature environments (26). DegP has also been associated in virulence in Salmonella typhimurium because *degP* mutants are unable to survive and replicate in host cell tissues due to oxidative stress (27). The protein DegP is regulated by the alternative sigma factor E (RpoE) which responds to extracellular stress, such as heat shock and other stressors that result in misfolded proteins (28, 29). The other divergent protein, RseP, is an integral membrane zinc-containing site-2 metalloprotease and is important in activating the sigma factor E (RpoE) by cleaving the anti-sigma factor RseA with DegS (21). Currently, the roles of RseP, DegP, and the alternative sigma factor in *Liberibacter* taxa are unknown; however, we hypothesize that these proteins may be important for the successful colonization of both psyllid and plant host tissues.

The Sec-dependent secretion systems in *Liberibacter* are hypothesized to be important in the secretion of pathogenesis factors, as *Liberibacter* does not encode a Type III secretion system (23). In this study, we found that SecG is highly divergent in *Ca*. L. capsica compared to other *Liberibacter* species (Table 3; Fig. S4). The protein SecG forms a complex with SecYE and stimulates translocation in bacteria, especially at low temperatures (30). Previous studies in *Liberibacter* have revealed that Sec-dependent proteins likely play a different role in their psyllid versus plant host environments. For example, differential expression of Sec-dependent complex proteins and effectors in *Ca.* L. asiaticus was observed between psyllid and plant host tissues (6, 23). In regard to the SecYEG complex, Yan et al. (23) revealed that *secE* and *secD/F* mRNAs were both upregulated in sweet orange (*Citrus sinensis*) compared to the Asian citrus psyllid (*Diaphorina citri*) (23). It will be of interest for future studies in *Liberibacter* to determine how amino acid mutations in Sec-dependent complex proteins, such as SecYEG complex, may impact *Liberibacter* pathogenesis and host-microbe interactions.

Shared and unique orthologous protein clusters were also identified among *Liberibacter* taxa in this study (Fig. 1). As expected, most of the shared orthologous protein clusters are single copy genes, primarily under purifying selection, that are important in both housekeeping functions and environmental response (Fig. 1B; Table S2 and S3). This phenomenon of widespread purifying selection for orthologous genes has been documented in many host-restricted insect endosymbionts with reduced genomes, such as *Buchnera*, *Blochmannia*, *Carsonella*, and *Wolbachia* (31–33), potentially because these genes are critical for the life and function of these microbes as mutualists or pathogens. Most orthologous gene cluster functions that are unique to each *Liberibacter* lineage have no known relatives and/or are hypothetical or unknown. It is currently unclear if this orphan gene status in *Liberibacter* lineages is due to limited sampling effort in databases, fast evolving genes, and/or if these genes recently formed *de novo*. Nevertheless, we identified an orthologous MFS transporter gene cluster in *Ca.* L. americanus that is unique to this *Liberibacter* lineage and appears to have been acquired from a donor that is closely related to *Wolbachia*. These results suggest that these genes may have been horizontally transferred from *Wolbachia* into *Ca.* L. americanus’s genome. Alternatively, the MFS transporter genes may have been lost from all known *Liberibacter* lineages but retained in *Ca.* L. americanus. Deeper sampling of related genomes can help resolve this evolutionary relationship. The *Wolbachia* lineages that were most closely related to these putative horizontally transferred MFS transporters are harbored by insect, nematode, and spider hosts (Fig. 3). These MFS transporters, such as ProP and OusA, are important in protecting cells from dehydration under hyperosmotic stress by importing proline and glycine betaine into the cells. As a result, *Ca.* L. americanus may be more robust to osmotic stress compared to other *Liberibacter* lineages.

In summary, the draft genome of the new *Liberibacter* species *Ca*. L. capsica was opportunistically derived from a limited amount of insect metagenomic material (5), and we further refined and annotated *Ca*. L. capsica’s draft genome here. Moreover, we estimated the rates of molecular evolution between *Ca.* L. capsica and five other *Liberibacter* species (*Ca.* L. asiaticus, *Ca.* L. psyllaurous, *Ca.* L. americanus, *Ca.* L. africanus, and *L. crescens*) to identify important protein candidates in *Ca*. L. capsica that may be involved in insect-plant interactions. Future research will extend the results from this study to further understand the genetics of *Ca*. L. capsica and, more broadly, the evolution of *Liberibacter* lineages.

## MATERIALS AND METHODS

### Identification of *Ca.* Liberibacter capsica contigs and proteins.

The metagenome of *Ca.* Liberibacter capsica was assembled previously in Kwak et al. (5) (accession number: SRR15069915). To further refine and annotate metagenomic contigs identified in Kwak et al. (5), we used Prokka v1.14.5 (34) to identify putative *Ca.* Liberibacter capsica proteins, followed by a BLASTP analysis against the NCBI nr database (downloaded on 04/2021) using DIAMOND v.2.0.13 (35) with an E value cutoff of 10e-10. Criteria for establishing if annotated proteins belong to *Ca.* L. capsica’s genome were the following: (i) if the putative *Ca.* L. capsica protein’s best hit was a *Ca.* Liberibacter species, this protein was binned as *Ca.* Liberibacter capsica; (ii) if the putative *Ca.* L. capsica protein’s best hit was identified as a gene from a genus other than *Liberibacter*, it was further determined if this protein was surrounded by other *Liberibacter* proteins (based on BLASTP results) on the same contig. If surrounding proteins on the same contig were identified as *Liberibacter* proteins, then this protein was binned as *Ca.* Liberibacter capsica; and (iii) if the putative *Ca.* L. capsica protein was the only gene on a contig and had a best hit to a bacterium that belongs to Alphaproteobacteria with a percent identity less than 95%, it was binned as a *Ca.* Liberibacter capsica candidate gene. The final contigs that possessed proteins that were binned as *Ca.* Liberibacter capsica were submitted to NCBI genome as a draft *Ca.* Liberibacter capsica genome (accession number: JAMJGA000000000). Emboss 6.6.0 (36) infoseq was used on the final draft of the *Ca.* Liberibacter capsica genome to determine total nucleotide length.

### Identification of orthologous clusters in *Ca.* L. capsica.

Protein orthologs were identified in *Ca.* L. capsica using all of the *Liberibacter* species with fully sequenced genomes that were available in NCBI. These species include *Ca.* L. asiaticus (NZ_CP019958.1), *Ca.* L. psyllaurous (also known as *Ca.* L. solanacearum) (NC_014774), *Ca.* L. americanus (NC_022793.1), *Ca.* L. africanus (NZ_CP004021.1), and *L. crescens* (NZ_CP010522.1). For standardizing protein and nucleotide coding sequence data sets for the analysis of orthologous genes among the latter *Liberibacter* species, we used Prokka v1.14.5 (33) to identify and annotate proteins and nucleotide coding sequences for each *Liberibacter* and *Ca.* Liberbacter species. Orthologous clusters of proteins from *Ca.* L. capsica, *Ca.* L. asiaticus, *Ca.* L. psyllaurous, *Ca.* L. americanus, *Ca.* L. africanus, and *L. crescens* were determined using OrthoVenn2 (37), using the default settings. Orthologous clusters were further assigned GO terms (38, 39) and Swiss-Prot terms (40, 41) using OrthoVenn2 (37), using the default settings. All *Ca.* L. capsica proteins assigned to clusters were annotated further using BlastKOALA (42), Revigo (43), and NCBI BLAST (16) against nr with an E value cutoff of 10e-10. For the phylogenetic analyses of putative horizontally transferred genes and highly diverging Tad pilus genes, sequences were retrieved from NCBI GenBank and from this study, and they were aligned with MAFFT v7.505 (44). Phylogenetic tree construction was conducted with RAxML v8.2.12 (45), using the GTR substitution model and default bootstrap parameters.

### Estimating rates of molecular evolution in *Ca.* L. capsica proteins.

To estimate the rates of molecular evolution between *Ca.* L. capsica and the other five *Liberibacter* species (*Ca.* L. asiaticus, *Ca.* L. psyllaurous, *Ca.* L. americanus, *Ca.* L. africanus, and *L. crescens*), we calculated the nonsynonymous substitutions per nonsynonymous site (dN) and the synonymous substitutions per synonymous site (dS) for one-to-one coding sequence orthologs from OrthoVenn2 (37). Similar to Degnan et al. (46), nucleotide sequences for each set of orthologous coding sequences were aligned using MAFFT (44). Then, all gaps and stop codons were removed, and pairwise estimates of (dN) and (dS) were calculated in Paml (47) based on the method of Goldman and Yang (48). Gene pairs were excluded when dS was saturated (dS ≥ 3.0).

### Data availability.

The draft genome assembly of *Ca.* Liberibacter capsica curated here was submitted to NCBI Genome under the accession number JAMJGA000000000. The raw metagenomic data of *Ca.* Liberibacter capsica can be found here (accession number: SRR15069915; [5]). The Prokka annotation GFF file and protein fasta files for the draft genome of *Ca.* Liberibacter capsica are in Table S5 and Table S6, respectively.

## References

[1] Lloyd KG, Steen AD, Ladau J, Yin J, Crosby L. 2018. Phylogenetically novel uncultured microbial cells dominate Earth microbiomes. mSystems 3:e00055-18. doi:10.1128/mSystems.00055-18. 30273414 PMC6156271

[2] Dubey A, Malla MA, Kumar A. 2022. Role of next-generation sequencing (NGS) in understanding the microbial diversity, p 307–328. In Kumar A, Choudhury B, Dayanandan S, Khan ML (ed), Molecular Genetics and Genomics Tools in Biodiversity Conservation. Springer, Singapore.

[3] Bové JM. 2006. Huanglongbing: a destructive, newly-emerging, century-old disease of citrus. J Plant Pathol 88:7–37.

[4] Wang N, Pierson EA, Setubal JC, Xu J, Levy JG, Zhang Y, Li J, Rangel LT, Martins J. 2017. The Candidatus Liberibacter–host Interface: insights into pathogenesis mechanisms and disease control. Annu Rev Phytopathol 55:451–482. doi:10.1146/annurev-phyto-080516-035513. 28637377

[5] Kwak Y, Sun P, Meduri VR, Percy DM, Mauck KE, Hansen AK. 2021. Uncovering symbionts across the psyllid tree of life and the discovery of a new Liberibacter species, “Candidatus” Liberibacter capsica. Front Microbiol 12. doi:10.3389/fmicb.2021.739763. PMC8511784 34659173

[6] Thapa SP, De Francesco A, Trinh J, Gurung FB, Pang Z, Vidalakis G, Wang N, Ancona V, Ma W, Coaker G. 2020. Genome-wide analyses of Liberibacter species provides insights into evolution, phylogenetic relationships, and virulence factors. Mol Plant Pathol 21:716–731. doi:10.1111/mpp.12925. 32108417 PMC7170780

[7] Fagen JR, Leonard MT, Coyle JF, McCullough CM, Davis-Richardson AG, Davis MJ, Triplett EWY. 2014. Liberibacter crescens gen. nov., sp. nov., the first cultured member of the genus Liberibacter. Int J Syst Evol Microbiol 64:2461–2466. doi:10.1099/ijs.0.063255-0. 24786353

[8] Wang N. 2019. The citrus Huanglongbing crisis and potential solutions. Mol Plant 12:607–609. doi:10.1016/j.molp.2019.03.008. 30947021

[9] Hansen AK, Trumble JT, Stouthamer R, Paine TD. 2008. A new Huanglongbing species, “Candidatus Liberibacter psyllaurous,” found to infect tomato and potato, is vectored by the psyllid Bactericera cockerelli (sulc). Appl Environ Microbiol 74:5862–5865. doi:10.1128/AEM.01268-08. 18676707 PMC2547047

[10] Munyaneza JE, Fisher TW, Sengoda VG, Garczynski SF, Nissinen A, Lemmetty A. 2010. First report of “Candidatus Liberibacter solanacearum” associated with psyllid-affected carrots in Europe. Plant Dis 94:639–639. doi:10.1094/PDIS-94-5-0639A. 30754456

[11] Haapalainen M, Wang J, Latvala S, Lehtonen MT, Pirhonen M, Nissinen AI. 2018. Genetic variation of ‘Candidatus Liberibacter solanacearum’ haplotype C and identification of a novel haplotype from Trioza urticae and stinging nettle. Phytopathology 108:925–934. doi:10.1094/PHYTO-12-17-0410-R. 29600888

[12] Sengoda VG, Munyaneza JE, Crosslin JM, Buchman JL, Pappu HR. 2010. Phenotypic and etiological differences between psyllid yellows and zebra chip diseases of potato. Am J Pot Res 87:41–49. doi:10.1007/s12230-009-9115-x.

[13] Duan Y, Zhou L, Hall DG, Li W, Doddapaneni H, Lin H, Liu L, Vahling CM, Gabriel DW, Williams KP, Dickerman A, Sun Y, Gottwald T. 2009. Complete genome sequence of citrus Huanglongbing bacterium, ‘Candidatus Liberibacter asiaticus’ obtained through metagenomics. Mol Plant Microbe Interact 22:1011–1020. doi:10.1094/MPMI-22-8-1011. 19589076

[14] National Center for Biotechnology Information. 2022. https://www.ncbi.nlm.nih.gov/. Retrieved 21 April 2022.

[15] Burckhardt D, Queiroz DL, Rezende MQ, de Queiroz EC, Bouvet JP. 2012. The capsicum psyllid, Russelliana capsici (Hemiptera, Psylloidea), a pest on Capsicum annuum (Solanaceae) in Argentina and Brazil. Mitteilungen Schweiz Entomol Ges 85:71–78.

[16] Camacho C, Coulouris G, Avagyan V, Ma N, Papadopoulos J, Bealer K, Madden TL. 2009. BLAST+: architecture and applications. BMC Bioinformatics 10:421. doi:10.1186/1471-2105-10-421. 20003500 PMC2803857

[17] Andrade M, Wang N. 2019. The tad pilus apparatus of ‘Candidatus Liberibacter asiaticus’ and its regulation by VisNR. Mol Plant Microbe Interact 32:1175–1187. doi:10.1094/MPMI-02-19-0052-R. 30925227

[18] Cai L, Jain M, Sena-Vélez M, Jones KM, Fleites LA, Heck M, Gabriel DW. 2021. Tad pilus-mediated twitching motility is essential for DNA uptake and survival of Liberibacters. PLoS One 16:e0258583. doi:10.1371/journal.pone.0258583. 34644346 PMC8513845

[19] Van Gijsegem F, Genin S, Boucher C. 1993. Conservation of secretion pathways for pathogenicity determinants of plant and animal bacteria. Trends Microbiol 1:175–180. doi:10.1016/0966-842X(93)90087-8. 8143135

[20] Krojer T, Garrido-Franco M, Huber R, Ehrmann M, Clausen T. 2002. Crystal structure of DegP (HtrA) reveals a new protease-chaperone machine. 6879. Nature 416:455–459. doi:10.1038/416455a. 11919638

[21] Akiyama Y, Kanehara K, Ito K. 2004. RseP (YaeL), an Escherichia coli RIP protease, cleaves transmembrane sequences. EMBO J 23:4434–4442. doi:10.1038/sj.emboj.7600449. 15496982 PMC526465

[22] Spencer J, Murphy LM, Conners R, Sessions RB, Gamblin SJ. 2010. Crystal structure of the LasA virulence factor from Pseudomonas aeruginosa: substrate specificity and mechanism of M23 metallopeptidases. J Mol Biol 396:908–923. doi:10.1016/j.jmb.2009.12.021. 20026068

[23] Yan Q, Sreedharan A, Wei S, Wang J, Pelz-Stelinski K, Folimonova S, Wang N. 2013. Global gene expression changes in Candidatus Liberibacter asiaticus during the transmission in distinct hosts between plant and insect. Mol Plant Pathol 14:391–404. doi:10.1111/mpp.12015. 23336388 PMC6638839

[24] Serbina L, Burckhardt D. 2017. Systematics, biogeography and host-plant relationships of the neotropical jumping plant-louse genus Russelliana (Hemiptera: psylloidea). Zootaxa 4266:1–114. doi:10.11646/zootaxa.4266.1.1. 28610391

[25] Clock SA, Planet PJ, Perez BA, Figurski DH. 2008. Outer membrane components of the tad (tight adherences Secreton of Aggregatibacter actinomycetemcomitans. J Bacteriol 190:980–990. doi:10.1128/JB.01347-07. 18055598 PMC2223556

[26] Strauch KL, Johnson K, Beckwith J. 1989. Characterization of degP, a gene required for proteolysis in the cell envelope and essential for growth of Escherichia coli at high temperature. J Bacteriol 171:2689–2696. doi:10.1128/jb.171.5.2689-2696.1989. 2540154 PMC209953

[27] Johnson K, Charles I, Dougan G, Pickard D, O'Gaora P, Costa G, Ali T, Miller I, Hormaeche C. 1991. The role of a stress-response protein in Salmonella typhimurium virulence. Mol Microbiol 5:401–407. doi:10.1111/j.1365-2958.1991.tb02122.x. 1645840

[28] Erickson JW, Gross CA. 1989. Identification of the sigma E subunit of Escherichia coli RNA polymerase: a second alternate sigma factor involved in high-temperature gene expression. Genes Dev 3:1462–1471. doi:10.1101/gad.3.9.1462. 2691330

[29] Meltzer M, Hasenbein S, Mamant N, Merdanovic M, Poepsel S, Hauske P, Kaiser M, Huber R, Krojer T, Clausen T, Ehrmann M. 2009. Structure, function and regulation of the conserved serine proteases DegP and DegS of Escherichia coli. Res Microbiol 160:660–666. doi:10.1016/j.resmic.2009.07.012. 19695325

[30] Dalbey RE, Chen M. 2004. Sec-translocase mediated membrane protein biogenesis. Biochim Biophys Acta BBA - Mol Cell Res 1694:37–53. doi:10.1016/j.bbamcr.2004.03.009. 15546656

[31] Tamas I, Wernegreen JJ, Nystedt B, Kauppinen SN, Darby AC, Gomez-Valero L, Lundin D, Poole AM, Andersson SGE. 2008. Endosymbiont gene functions impaired and rescued by polymerase infidelity at poly(A) tracts. Proc Natl Acad Sci USA 105:14934–14939. doi:10.1073/pnas.0806554105. 18815381 PMC2567471

[32] Woolfit M, Iturbe-Ormaetxe I, McGraw EA, O'Neill SL. 2009. An ancient horizontal gene transfer between mosquito and the endosymbiotic bacterium Wolbachia pipientis. Mol Biol Evol 26:367–374. doi:10.1093/molbev/msn253. 18988686

[33] Sloan DB, Moran NA. 2012. Genome reduction and co-evolution between the primary and secondary bacterial symbionts of psyllids. Mol Biol Evol 29:3781–3792. doi:10.1093/molbev/mss180. 22821013 PMC3494270

[34] Seemann T. 2014. Prokka: rapid prokaryotic genome annotation. Bioinformatics 30:2068–2069. doi:10.1093/bioinformatics/btu153. 24642063

[35] Buchfink B, Xie C, Huson DH. 2015. Fast and sensitive protein alignment using DIAMOND. 1. Nat Methods 12:59–60. doi:10.1038/nmeth.3176. 25402007

[36] Rice P, Longden I, Bleasby A. 2000. EMBOSS: the European Molecular Biology Open Software Suite. Trends Genet 16:276–277. doi:10.1016/s0168-9525(00)02024-2. 10827456

[37] Xu L, Dong Z, Fang L, Luo Y, Wei Z, Guo H, Zhang G, Gu YQ, Coleman-Derr D, Xia Q, Wang Y. 2019. OrthoVenn2: a web server for whole-genome comparison and annotation of orthologous clusters across multiple species. Nucleic Acids Res 47:W52–W58. doi:10.1093/nar/gkz333. 31053848 PMC6602458

[38] Ashburner M, Ball CA, Blake JA, Botstein D, Butler H, Cherry JM, Davis AP, Dolinski K, Dwight SS, Eppig JT, Harris MA, Hill DP, Issel-Tarver L, Kasarskis A, Lewis S, Matese JC, Richardson JE, Ringwald M, Rubin GM, Sherlock G. 2000. Gene ontology: tool for the unification of biology. The Gene Ontology Consortium. Nat Genet 25:25–29. doi:10.1038/75556. 10802651 PMC3037419

[39] Gene Ontology Consortium. 2021. The Gene Ontology resource: enriching a GOld mine. Nucleic Acids Res 49:D325–D334. doi:10.1093/nar/gkaa1113. 33290552 PMC7779012

[40] Boutet E, Lieberherr D, Tognolli M, Schneider M, Bairoch A. 2007. UniProtKB/Swiss-Prot. Methods Mol Biol Clifton NJ 406:89–112. 10.1007/978-1-59745-535-0_4 18287689

[41] Boutet E, Lieberherr D, Tognolli M, Schneider M, Bansal P, Bridge AJ, Poux S, Bougueleret L, Xenarios I. 2016. UniProtKB/Swiss-Prot, the manually annotated section of the UniProt KnowledgeBase: how to use the entry view, p 23–54. *In* Edwards D (ed), Plant Bioinformatics: Methods and Protocols. Springer, New York, NY. 10.1007/978-1-4939-3167-5_2 26519399

[42] Kanehisa M, Sato Y, Morishima K. 2016. BlastKOALA and GhostKOALA: KEGG tools for functional characterization of genome and metagenome sequences. J Mol Biol 428:726–731. doi:10.1016/j.jmb.2015.11.006. 26585406

[43] Supek F, Bošnjak M, Škunca N, Šmuc T. 2011. REVIGO summarizes and visualizes long lists of gene ontology terms. PLoS One 6:e21800. doi:10.1371/journal.pone.0021800. 21789182 PMC3138752

[44] Katoh K, Standley DM. 2013. MAFFT multiple sequence alignment software version 7: improvements in performance and usability. Mol Biol Evol 30:772–780. doi:10.1093/molbev/mst010. 23329690 PMC3603318

[45] Stamatakis A. 2014. RAxML version 8: a tool for phylogenetic analysis and post-analysis of large phylogenies. Bioinformatics 30:1312–1313. doi:10.1093/bioinformatics/btu033. 24451623 PMC3998144

[46] Degnan PH, Leonardo TE, Cass BN, Hurwitz B, Stern D, Gibbs RA, Richards S, Moran NA. 2010. Dynamics of genome evolution in facultative symbionts of aphids. Environ Microbiol 12:2060–2069. doi:10.1111/j.1462-2920.2009.02085.x. 21966902 PMC2955975

[47] Yang Z. 1997. PAML: a program package for phylogenetic analysis by maximum likelihood. Comput Appl Biosci 13:555–556. 9367129 10.1093/bioinformatics/13.5.555

[48] Goldman N, Yang Z. 1994. A codon-based model of nucleotide substitution for protein-coding DNA sequences. Mol Biol Evol 11:725–736. doi:10.1093/oxfordjournals.molbev.a040153. 7968486

