# Supplementary material for: Divergent Host-Microbe Interaction and Pathogenesis Proteins Detected in Recently Identified Liberibacter Species
Source: Microbiol Spectr. 2022 Jul 28;10(4):e02091-22. doi: 10.1128/spectrum.02091-22 (PMC9430466; doi:10.1128/spectrum.02091-22)
Supplement: Supplemental file 1 — Fig. S1 to S4. Download spectrum.02091-22-s0001.pdf, PDF file, 0.2 MB [file spectrum.02091-22-s0001.pdf]

Supplemental Figures
Figure S1- S4

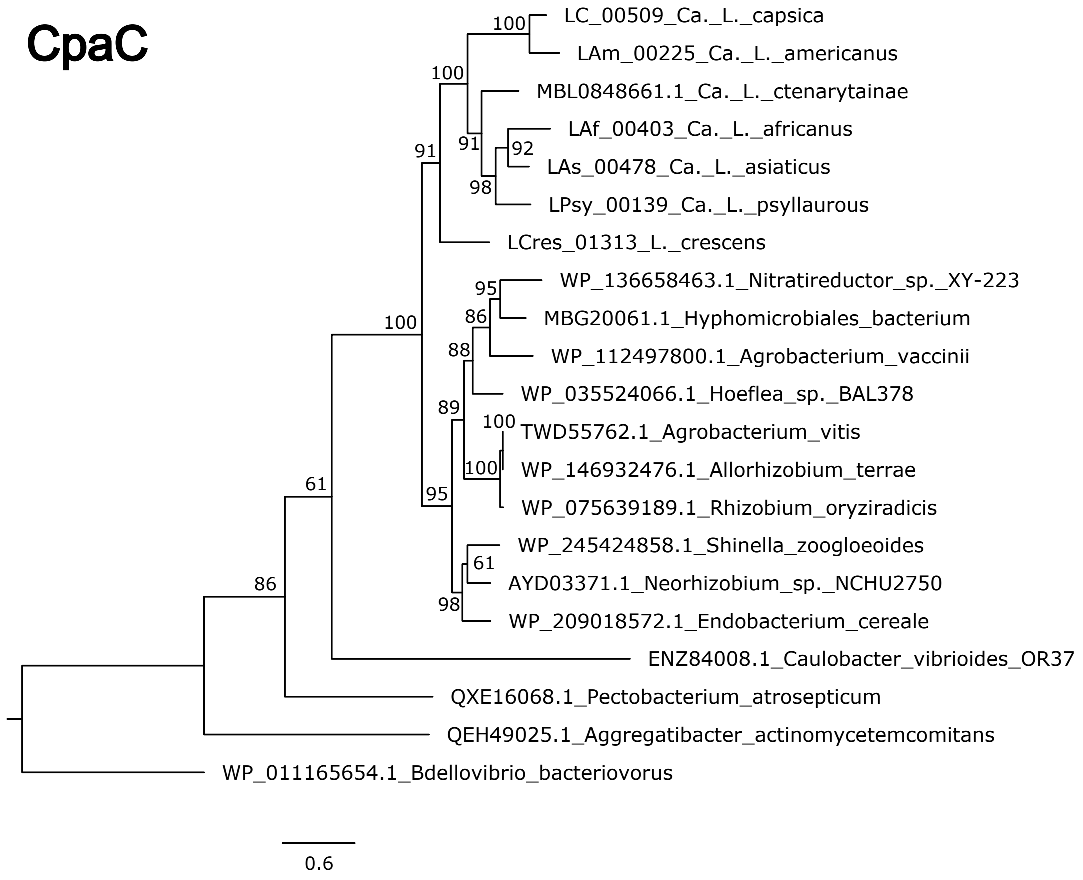

Figure S1. Phylogenetic relationship of pilus assembly protein, secretin CpaC, among *Liberibacter* and other bacterial representatives. The abbreviations for each *Liberibacter* species locus number are LC=Ca. L. capsica, LAs= *Ca.* L. asiaticus (NZ_CP019958.1), LPsy= *Ca.* L. psyllaurous (NC_014774), LAm= *Ca.* L. americanus (NC_022793.1), LAf= *Ca.* L. africanus (NZ_CP004021.1), and LCres= *L. crescens* (NZ_CP010522.1). To standardize protein and nucleotide coding sequence datasets for the analysis of orthologous genes among the latter *Liberibacter* species we used Prokka v1.14.5. Gene numbers after species abbreviations are numbers assigned by the Prokka pipeline. Bootstrap values are indicated for nodes with 50% or above. The tree was rooted with an outgroup *Bdellovibrio Bacteriovorus* (WP011165654.1).

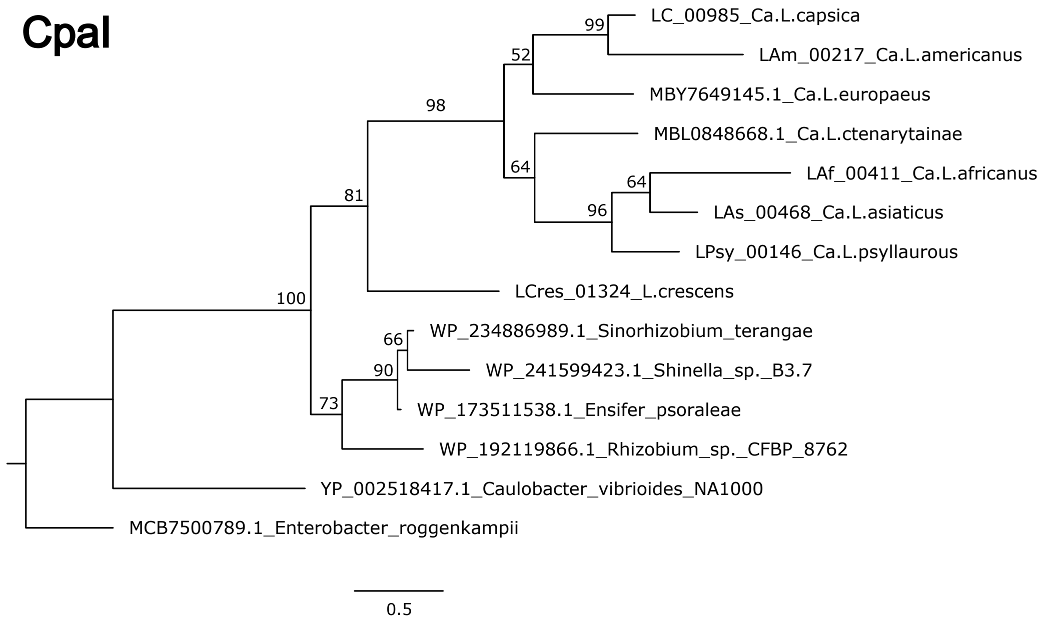

Figure S2. Phylogenetic relationship of the N-terminal domain-containing pilus assembly protein, CpaI, among *Liberibacter* and other bacterial representatives. The abbreviations for each *Liberibacter* species locus number are LC=Ca. L. capsica, LAs= *Ca*. L. asiaticus (NZ_CP019958.1), LPsy= *Ca*. L. psyllaurous (NC_014774), LAm= *Ca*. L. americanus (NC_022793.1), LAf= *Ca.* L. africanus (NZ_CP004021.1), and LCres= *L. crescens* (NZ_CP010522.1). To standardize protein and nucleotide coding sequence datasets for the analysis of orthologous genes among the latter *Liberibacter* species we used Prokka v1.14.5. Gene numbers after species abbreviations are numbers assigned by the Prokka pipeline. Bootstrap values are indicated for nodes with 50% or above. The tree was rooted with an outgroup *Enterobater roggenkampii* (MCB7500789.1).

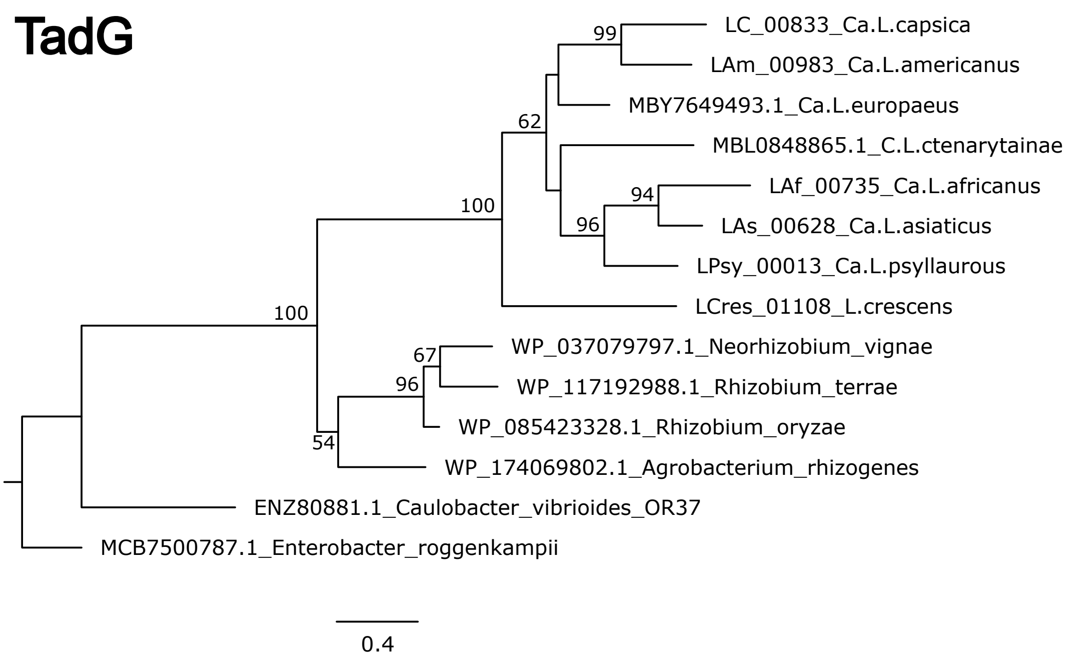

Figure S3. Phylogenetic relationship of pilus assembly protein, TadG, among *Liberibacter* and other bacterial representatives. The abbreviations for each *Liberibacter* species locus number are LC=Ca. L. capsica, LAs= *Ca*. L. asiaticus (NZ_CP019958.1), LPsy= *Ca*. L. psyllaurous (NC_014774), LAm= *Ca*. L. americanus (NC_022793.1), LAf= *Ca.* L. africanus (NZ_CP004021.1), and LCres= *L. crescens* (NZ_CP010522.1). To standardize protein and nucleotide coding sequence datasets for the analysis of orthologous genes among the latter *Liberibacter* species we used Prokka v1.14.5. Gene numbers after species abbreviations are numbers assigned by the Prokka pipeline. Bootstrap values are indicated for nodes with 50% or above. The tree was rooted with an outgroup *Enterobater roggenkampii* (MCB7500787.1).

Figure S4. Alignments of the 21 divergent *Liberibacter* proteins. The abbreviations for each *Liberibacter* species locus number are LC=Ca. L. capsica, LAs= *Ca*. L. asiaticus (NZ_CP019958.1), LPsy= *Ca*. L. psyllaurous (NC_014774), LAm= *Ca*. L. americanus (NC_022793.1), LAf= *Ca.* L. africanus (NZ_CP004021.1), and LCres= *L. crescens* (NZ_CP010522.1). To standardize protein and nucleotide coding sequence datasets for the analysis of orthologous genes among the latter *Liberibacter* species we used Prokka v1.14.5. Gene numbers after species abbreviations are numbers assigned by the Prokka pipeline. Gene description based on top Blastp hits is provided before each alignment.

```
CLUSTAL O(1.2.4) multiple sequence alignment

1 pyruvate dehydrogenase complex dihydrolipoamide acetyltransferase

LCres_01135     -MTIKITMPALSPTMEEGKIAKWLVKEGDSVSSGDIICEIETDKAIMEVESVYEGIVEEI      59
LPsy_00459      -MINTITMPSLSPTMKTGKLAKWLVKKGDKIYPGDIICEIETDKAIMEFESVDEGVVHEI      59
LAf_00305       -MIHTITMPSLSPTMKTGSIKKWKIKKGDKIFPGDILCEIETDKAIMEFESVDEGAVHEI      59
LAs_00528       MMIHTITMPSLSPTMTEGKLAKWIKQEGDKISPGDILCEIETDKAIMEFESVDEGIIDEI      60
LC_00045        -MINTITMPALSPTMEEGKLEKWLVKKGDSFKPGDVLCEIETDKAIMEVEAVDDGYVHEI      59
LAm_00317       -MKHTINMPALSPTMEEGKLERWSVKKGDCFKPGDIICEVETDKAIMDVEAVDEGCVHEI      59
                 *  .*.**:*****  *.: :*  ::**  .  **::**:*******: *:* :* :.**

LCres_01135     TVPEGTENVKVNSVILLLSGEDDSDISEPSNISQTQHPSSSPEEILSNPLSRENSAELNQ      119
LPsy_00459      LTSEGTENIKVNSPILNILIDCDGGAPAPILP--EKNFVEIEKESSDPAIS-SFA-----  111
LAf_00305       IALEGTENIPVNSPILSIVMDYAPP---PTPA--KDNPVEVEKGFSDIPLI-SLE-----  108
LAs_00528       LVPAGTENIAVNSPILNILMDSTEIPPSPPLS--KENIVEVREEHSHSS---PVV-----  110
LC_00045        IVPEKTEHIKVNSPILTFISDNDVKLS-NSKP--QVDHIESTNESPASPSS-SNRPTINQ      115
LAm_00317       LVPEKTEHIKVNTPILTFISDGDITSSSLTTP--KVDHIESKSESFTSTAN-SNISNIKK      116
                .   **:: **: ** :  :             .  .  .

LCres_01135     --LEISINKEKVFSSPLARRIAKEKNIDIFSIKGSGPYGRVIKRDVENSLPTNHDILNIK      177
LPsy_00459      PTEKKVCGQDFPASSPLARRLAKENGIDLSSVSGSGPRGRIVKNDIEQLILHNTGVKHAS      171
LAf_00305       K--QKSHRKNSPISSPLARRLAKEHGIDLSSLSGSGPHGRIIKDDVKSFIAQKTNVNKFP      166
LAs_00528       V--REKHSKNRPIASPLARRLAGEHGIDLSSLSGSGPHGRIVKSDIETLISTKTNVKDYS      168
LC_00045        PENQFLQRINHIPSSPLARRLASEHKINLASVSGSGPNGRIIKKDIEHFIENGSNE-YSS      174
LAm_00317       AEDKIVQHVNHINSSPLARRLASEYEIDLASISGSGPNGRIIKKDIERFIENSFPNKYLS      176
                .    :   :******:* *  *:: *: **** **::* *::  :

LCres_01135     A-PSSSFGS-GLMPDASILKLFEKGSYDLEPHDSMRKTIAARLQQATQTIPHFYVSIDCE      235
LPsy_00459      TAQSASIESMNSSVDDDIMRLFAPNSYEIIPHDNMRKTIASRLQQSKQTIPHFYVSIDCN      231
LAf_00305       STQ--NFESINTSVDDDIIKIFAEGSYEVIPHDNMRKTIAYRLQQSKQTIPHFYVSIDCN      224
LAs_00528       TIQ--SFGLVDESIDANILNLFAKDSYEVIPHDNIRKTIACRLQQSKQTIPHFYVSIDCN      226
LC_00045        RIKKYS----------------------------------------------------180
LAm_00317       GMTNPDVKE-AEISSDDINHLFAKDSYEINYHNNMRKTIANRLLQSKQNIPHFYVSIDCN      235
                 .

LCres_01135     IDQLLALRSQINTSISIHNDQSSIKISVNDMVIKALAMSMLKVPSANVSWTETALIKHHH      295
LPsy_00459      IDNLLSLRQQMNLFAQSNKKENINKISVNDVILKAFSLAMLQVPEANVSWTTNALIRHKN      291
LAf_00305       IDNLLSLREQMNLIIQANTKDTPNKISVNDVIIKAFALAMTKVPEANVSWTTNALIQHKN      284
LAs_00528       IDNLLSLREQMNRTLQFHREEISNKISVNDIILKAFALAMIQVPEANVSWTTNAMIRHKH      286


CLUSTAL O(1.2.4) multiple sequence alignment

trypsin-like peptidase domain-containing protein, DegP

LCres_01279     MFKIKNQFMSSKFFFKFLSIALLAMVVSVSGWPIKIADSFALPVISGPQIPSFAPVVDAV      60
LC_00293        MLKTKTL--PIRFFPLI---VAITCAF------FGGS-YSIIEARD-SLYFDISPAVERV  47
LAm_00252       MLKIQNL--PIIIFSLV---AVLIFSV------FGGSIRSVVEARD-ASYVDISPVVDRV  48
LPsy_00112      MFKSHIL--SIRNISIT---V--LMYI------FFASTCFVAEARIP-PSTDLVPIVERV  46
LAf_00376       MFKINIL--SIRSVFVS---ALILMFV------IMGCLCFAAEARV-VPGVDLSTVVARV  48
LAs_00505       MFKRQIL--SVKSICTV---ALTCV--------IFSSTYLVLEAKLPPSSVDLPPVIARV  47
                *:* :      .    .          :  .      .:   :  *

LCres_01279     YSSVVSVRVQSKLSPVSD-KEMELFDFYGFDDLPDDHPLSRFFRDFG-LRKKTEKKSKNA      118
LC_00293        SPSVVTVVVKTSLNLHDVFRPKSPIDGERYQNVP---------SPVFNRFPRDP------  92
LAm_00252       ASSVVTVVVKIKKS-----NNIGVIGSAQYQNLP---------SLVLSKFPHISDYISGK  94
LPsy_00112      SRSVVSILVEPKKEVLEK----EFLKLYGFDTLPDSNPLKNYLHN----FFSNEGNESAG      98
LAf_00376       SPSVVSVFIKPKKKLLEK----ELLEFYGFGNLPEDHPLRHYFQN----FLPDEGRGSSD      100
LAs_00505       SPSIVSVMVEPKKKVSVE----QMFNAYGFGNLPEDHPLKNYFRKDFHKFFSGEEPILSD      103
                *:*:: :: . .        :    :  :*

LCres_01279     PLRPIAQGSGFFVSEDGYIVTNNHVIADGSS---FIIVLNDGTELPAKLVGKDTRTDLAV      175
LC_00293        ---GTILGAGFFISADGYVVTNSHVVADGDGISDISVVLKGGETLPAVFIGNDSVTDLAV      149
LAm_00252       DVKDVIIGSGFFISTDGYVVTNSHVVKNGADIAGIDVVLKGGAILPAYLVGYDHITDLAV      154
LPsy_00112      ITEKLASGSGFFVTENGYVLTSNHVVERGVS---FSVVLSDNTEFPAKVIGTDPMSDLAV      155
LAf_00376       RLESVTAGSGFFITDDGYIITNNHVIEDGAS---FSVVLSDETELPAQIVGTDIFSDLAI      157
LAs_00505       TVERLMFGSGFFITDDGYILTSNHIVEDGAS---FSVILSDDTELPAKLVGTDALFDLAV      160
                  *:***:: :**::*.:*::  *  .  : ::*..   :** .:* *   ***:

LCres_01279     LKVNDKRKFVYAEFGD-DNVRVGDWVLAVGNPFGLGGTVTAGIVSNRSRHI--GSGIYDD      232
LC_00293        LKVKSDKKFRAVEFGDTDSLRIGERVFAIGTPLGMSETVTSGIVSARGRSDFPTNKHGSS      209
LAm_00252       LKVKSDGKFNSVKFGDTDSVRIGERVFAIGSPLGISETVTSGIVSARGRSDFPTNKYGAS      214
LPsy_00112      LKVQSNQKFVPVKLEDSGNVHVGETVFTIGDPLGFRGSVNAGIVSARDRVL---ADKSGS      212
LAf_00376       LKVKSDKKFVPVEFEDMSNVRLGETVFTIGNPLGLRGSVNAGIVSALDRYL---SGKPGS      214
```

```
LAs_00505       LKVQSDRKFIPVEFEDANNIRVGEAVFTIGNPFRLRGTVSAGIVSALDRDI---PDRPGT          217
                ***:.. **  .:: * ..:::*: *:::* *: :  :*.:**** .*

LCres_01279     YIQIDAAVNRGNSGGPTFNLKGQVIGVNTAIVSNS-GGTNLGIAFAIPASTAKEVVSVLI          291
LC_00293        YLQVDVPITVGNSGGPCFNSSGRLVGVINYAFSAGN-GFGATIGFAIPTSVIKQVVPSLI          268
LAm_00252       YLQIDAPLTIGNSGGPCFNSNGLLVGINYYGFNV------SGIGFAISTSVIKEVIPSLI 268
LPsy_00112      YIQIDAPINQGNSGGPCFNTSGNVIGVSDIIITNNESASNVGIGLIVPVSVIKKVLPSLI          272
LAf_00376       FIQIDAPINKGNSGGPCFNSSGRVIGVNNLILTMGSSNANMGIGFIVPASTVKKSIPSLI          274
LAs_00505       FTQIDAPINQGNSGGPCFNALGHVIGVNAMIVTSG--QFHMGVGLIIPLSIIKKAIPSLI          275
                : *:*. :. ****** **  * ::*:    ..        ::: :  *  *: :  **

LCres_01279     KKGKIDRGWLGVHIQNVTKEIAESLGFAEQKGVFIAGVEKDSPAGKYGIKEGDIISAIEN          351
LC_00293        KNGKLIRGWTGLSFQNLTQNLAIPLGIKDKTGVIVVSVTKDSPADKIGLKIGDVICEING          328
LAm_00252       KNGRFIRGWSGISIQNLTKDLAVSFGIPDKTGVVVFSVTKDSPADKIGLKIGDVICELNG          328
LPsy_00112      EKGFVDRGWMGVVVQNLTQELARPLGLKGGNGLLVSSTVKDSPSDRAGIKPGDVICAIDG          332
LAf_00376       SKGSVDHGWVGLHMQNLTQELATPIGLKGTKGFLVLSVVTGSPADKVDIQEGDVICALNG          334
LAs_00505       SKGRVDHGWFGIMTQNLTQELAIPLGLRGTKGSLITAVVKESPADKAGMKVGDVICMLDG          335
                .:*  . :** *:  **:*:::*  :*:   .* .: .. .  **::  .:: **:*. ::

LCres_01279     VPVKDVRDLALRVASLPPGKKVELTVWHAGQEKKSVFVKLGTFPTDPEKEIKSTKEEEDK          411
LC_00293        KPIDNSLTFMKHINIYFPKDKIEFSLCSAKG-KRSVSLVLEASPTDTLMM-E----SRTL          382
LAm_00252       KPISNLMSFIKNINLYLPKDKIEISLCSEKG-KRSVSLVLEASPKDTLVL-E----SKRM          382
LPsy_00112      QIIKNTHDFVGRILSHSSKDKVEIRLCKEGG-DRSVPVYLESYPLVKEGD------AKQL          385
LAf_00376       KEIKDVQHFAWQISSRSPKEQVELSLCKEGN-KRSITVVLGTYPVEKNDVRS----ERKL          389
LAs_00505       RIIKSHQDFVWQIASRSPKEQVKISLCKEGS-KHSVAVVLGSSPTAKNDMHL----E---          387
                  :..   :  .:      .:::: :      .:*: : * : *

LCres_01279     GEGREISGLGIKVISDNSASGGLSITSVDENS--EAAEKGIKEGMRIISVNNSKVSSVED          469
LC_00293        QGTKELLGLKLKDVVYDDGRKAVRVVDANISS--EAYMKGVSPGMYIGYINSNQVSSIDD          440
LAm_00252       TDIKELLGIKMKDVIYEDGRKVVRIVDVNLSS--DAYFKGALPGMYISYINSSPVSSTDD          440
LPsy_00112      LATKKILGLRLQDFNYGT-KKIVRIVDMDLGA--EAVRKGIQKGMNIVSVNTNQVSCIKD          442
LAf_00376       SGTKELLGMILQDVHDGD-KKTVRIVSLDSNL--EAEQKGIQKGMDIISVNTKQVVCIKD          446
LAs_00505       VGDKELLGMVLQDINDGN-KKLVRIVALNPNREREVEAKGIQKGMTIVSVNTHEVSCIKD          446
                  ::: *: :: .       : :.  : .   :.  **    ** *  :*.  * . .*

LCres_01279     IEKILNLSRKKGRKN--ALFQIEFK-----------NGSNFIPLKIIKE-       505
LC_00293        VKRSIDEAKAKNNKFVVMLLQVNLDRDNTLRNKTWVNKDRFIDLAIN---              487
LAm_00252       VKRLIYEAKSKKNKSVSILLQTSPDRDDKLNNKTWIDKDGFIDVDID---              487
LPsy_00112      IESLIEQAREKNRDT--ILLQLSVDNDAKFNSNFYKNNVFFVSLKIKNIS              490
LAf_00376       VELLIRQAKEKKTDS--VLLKVKDSSGDVRPNNKIDNAGWFVSLKIK---              491
LAs_00505       VERLIGKAKEKKRDS--VLLQIKYDPDMQS---GNDNMSRFVSLKIDK--              489
                ::  :  :: *  .    *:: . .          :   *: : *
```

CLUSTAL O(1.2.4) multiple sequence alignment

hypothetical protein

```
LCres_00960     ---------------MIRTFLIGLWVSIVTLATVYVVLYMSVPDKP-----NESEDIGIK        40
LPsy_00543      MPIAFIPIDMRDFLIYVEFFFSGIWISIITIASFYILFLRSVDPQI----ESSKPLTTEQ    56
LAf_00789       ---------------MLKILFSGIWISIVTLISFYMLFIRSIDTNT----GNDASAV-IN        40
LAs_00689       ---------------MLKFLFSGIWISVVTLISFYMLFLRSMDTMV----ENHVPPLAIK        41
LC_00324        ---------------MLKIFLPGLWVSIVTILSFYVLFLRPSYTNT----ETYTQISEDK        41
LAm_00400       ---------------MLKIFLYGSWLLVVTLLSFYILFILPAYNNTNTNVENYTQITKDN        45
                               :. :: * *: ::*: :.*:::            .      :

LCres_00960     MSEILKSEQVSVPVISEGDVQAYFVVQLSFMIEKDKAKKINFFLKEMITDHLYTLLIGSS          100
LPsy_00543      SIHMITGELVAIPSISNGILQAYFFVKLSFIVNDRQSK---YYLKEISTDYLYTLLSGPP          113
LAf_00789       KNDMITGELVSIPVVSNGVIQAYFFIKLSFIVKSSQQR---LYLKAISTDYLYTLLAGSP          97
LAs_00689       NTNIIKSELVSIPSVSNGVLQAYFLVKLSFIVNDSQER---SYLKEIATDYLYTLLSGPP          98
LC_00324        SAYVIKGDLVTIPIIVDDVI-SYFFVKLSFIVDRSNKPESYNYFTDIATDYIYTLLSGSQ          100
LAm_00400       SNYVIRGDLITIPVISNGII-SYFFLKLSFVVDISNKPEYYNHFTDISTDYIYTLLAGSQ          104
                  :: .: :::* : :. : :** ::***::.  :      .:. : **::**** *

LCres_00960     VINIQKLKEFDLENFRKEVKEGLNSQFGTDVLLRVFIDKIDYFPKASIRHNSSRSKYRRI          160
LPsy_00543      MGDIVQIKSFGMDNFRKKIKEDLNLKLGSQFISDVLINELNYLSIADIRLNCFHLSDKES          173
LAf_00789       FGDLVQIKAFGFDNLRQKIKEDLNLKLGSPLILDVLIDKLHYLSIVDIRLNCWHLDSKAL          157
LAs_00689       MGDFVQIKAFGFDNLRKKIKEDLNSRLGSQFISEVLIDELNYLSIVDMRSNCLRLGSNAS          158
LC_00324        MSDFTKIKSFGVDNLRQTIKNDLNLKLGTKFLLDVLVTQFNYVSLNDIHTHCVNDGYKIP          160
LAm_00400       MGDLTKIKSFGLDNLRQKIKDDLNLKLGTKFLLEVLVNQFNYISLDGKQMSCVSEVYQVP          164
              . :: ::* *..:*:*: :*: ** ::*: .:  *:: ::.*.   . :  .     .

LCres_00960     KLLKFSESENFNTASP--        176
LPsy_00543      NLMAQKKSLLDKAKDKSQ        191
LAf_00789       DLITHKGSLIEKNKENLK        175
LAs_00689       DLMTQKGSLIEKNKEPLK        176
LC_00324        DLINDSRSVR-QKYDNN-        176
LAm_00400       DLRLGVDPDYVNKIDNRN        182
              .*        :  .
```

CLUSTAL O(1.2.4) multiple sequence alignment

PAS domain S-box protein

```
LCres_00213     RSRRLIKQPILIQNNIMELPL-SSETETNPLHSLKELTKEISISSLENFDTGNKDNLQTQ          239
LC_00401        ----------------MSIDYINSKTKNIIAKDRTDTKSTLSTRDGKD-------TS--I          35
LAm_00040       ----------------MSIDYINSKTKNISDKNCTDTKNNTSEIDIKS-------KS--I          35
LPsy_00383      ----------------MSIDSITLNPPNSPTNNCKNTSKDLSLKETIN-------NL--G          35
LAf_00635       ----------------MSIDSIISDSKNI-ISNCKDNTKDD---------------R--A     26
LAs_00286       ----------------MSTDSIISNPEDILTSNCKGNTQDLSKKKIDN-------GF--E          35
                      *.     .      . .  ..

LCres_00213     ITGMNDQEQTPETLSEEIKMQSQQSTEFESMKINTIDHLNQEIDNKTPFTFNAQPRVVRF          299
LC_00401        TTDFNAENATNKEFLKELSLIGNK-KN---L-EMNISCYEKKSESKNEFVFDPKPRTIRF          90
LAm_00040       TTDFNVENAINKESLKELPIIDSK-KK---I-EIDMHTSGEKSESKNEFLFDPKHSTVRF          90
LPsy_00383      IRDSHKGNPCNQNHLEQSSPTGQN-HA---PRT--QISCSRNLEPENSFLFDSSPRTVRF          89
LAf_00635       VKSSKKTNPCHDSL-EKSNFTNQR-RD---LGNVLLHDNNKNPGSTNAFVFDSMPRTVRF          81
LAs_00286       TEKSKKENPCHDSF-EQSNFTDQN-QE---SV-----SNTNYLDSENTFVFDPIPRTVRF          85
                  :  :   .  ::    ...              .     . * *:    .:**

LCres_00213     VWKVNAQTCFCEISKELPETIGPYAASIIGINFNDLNVILNLDPEGHIPELLKQQNTWSI          359
LC_00401        TWKINAEGYLTDISKELSQTIGEYTLKVIGMSFCDLNNLFRIDPNDYLSEVLKQQNTWYG          150
LAm_00040       TWKINAEGYIVDISKEFPQTIGQYTHKVVGMRFCDINNLLRIDPNNYLYEILKQQNTWYG          150
LPsy_00383      TWKIDAQGHLNAVSEELSQTIGTYALKMIGMHLCDINHILQIDSKDHLFDLLTQQNTWYS          149
LAf_00635       TWKLDASGYIREISEELSKAIGPYTFKMIGVRLCDINHILRIDPDNHIVDLLQKQNTWYG          141
LAs_00286       TWNIDAHGYLKEISEELPKTIGNYAFKMIGMRLCDVSDILHIDPNNHIGDLLKRQNTWYG          145
              .*:::*   :  :*:*: ::** *:  ::*: : *:  :: :*  ..: ::* :****

LCres_00213     ENTYWPIENTDLKVPIDFAALPTYTRDRKFDGFRGFGIIRISEAITDSLKTGLMINNIFN          419
LC_00401        KTTFWPIEGTKLHVPIDLSALPMYSQNHEFKGFKGLGIINIGQATNDPHQLGTTLDSIFS          210
LAm_00040       KTTFWPIEGTKLHVPIDLSALPMYSQNHEFKGFKGLGIINISQAKNDPHQFGTKLDSIFS          210
LPsy_00383      KTTFWPIEGTDLHVPIDLAALPIYSRDREFVGFKGFGIIRVNQTKNDPKKLGTTLGEKFS          209
```

```
LAf_00635      KNTIWPIEGTELHVPIDLAALPIYSRDREFTGFKGFGIVHVNRVSKDSHALGKILEEKLS      201
LAs_00286      KTTLWPIEGTNLYVPIDLAALPIYSRNREFSGFRGFGIVHVNRVDNDPRALGKRLDKKFS      205
               :.* ****.*.* ****::*** *:::::* **:*:**:.:.... .*    *  : . :.

LCres_00213    SLAEKIKVKYSYETNANLPQDTSFSLIPKEIKDKKELSLLEQQNP--------F------ 465
LC_00401       KLNNNGADNSFVKKY-ERACTS-SKNIP--FRDKNPFYTTKEQSSTANHVAQEFPKHTIS      266
LAm_00040      ESHNKTEDKSAIKKY-EHPIAS-SKKVP--LCSKPTIHTIKEQRYIENNTDLEFSKHTIS      266
LPsy_00383     QSHEIKKVHSFIKKE-NSPSLP-Y---------------------------------QT 233
LAf_00635      SSSEVKKDLSFVGQE-CFDLLS-K---------------------------------SS 225
LAs_00286      HLHEIKKGHSSVEKE-KYDIFS-Q---------------------------------QS 229
                  :

LCres_00213    LSNSDDISEKIILFSKYQMLNFDTLSHSGK-DALEDSSNHLRKNDNSDVLNSTPNTNTHN      524
LC_00401       FPANLDVKNKMAFISEYYLAKEDILTPTKKSSYSEDEDFCSINNDIVHTI---------- 316
LAm_00040      FPENLDVKNKMEFIYKHYLTKRDILKSAKQLSYSVDEPSFTVNNDSIHTI---------- 316
LPsy_00383     IPAHLDIKDRGSFPSGYFPKDKDDNLA-------TNKKNYFSKRDNFHTI---------- 276
LAf_00635      SQIHLSKKNKVSFPPQHYYDKD-DALKITKYPLFKDEENRIFNQENFHTI---------- 274
LAs_00286      PPPHLRMKNKVSSLTEYYAHKD-DVLKTEKYPLLTSEESSLPEQEDFHTI---------- 278
                   .::    :  .          ..   :.:  ..:

LCres_00213    KQINTQ-ETTALHQPIEKNQSSQKETFKYKSNCSVKLPKWVHEGYGLSVDNIDSISIPLL      583
LC_00401       -NLNKYTKDIKLK--LNSTNNEYEESFHYS---HPALSTYFIEEQNLTPEHADKYHIPLL      370
LAm_00040      -NLNTYVESIKPQ--INTIDDEYEKAFHYR---HYSLSTYFGENKNLTPEHADKYHIPLL      370
LPsy_00383     -NLNQYIEKYP------HPKESFEDSFQIN---HPSLSTYFGEGENLTPEIVDKHPISLF 326
LAf_00635      -NLNQYINKKHFNTPKNNNQK-SENLFHCN---HPSLSEYFAEGENIVPETIEKCPIPFC      329
LAs_00286      -NLNQYTKKQYFGTLQNNSKESFEYLSHRN---HPSLSAYFDEGENLTPETVDKCPIPFF      334
               ::*  :         ..  :  :      *  :. *  .:  :  :.  * :

LCres_00213    IYSGHRIYYTNLAFLILTGYKSHEEIETAGGLSKLLEPQHSQKNSEHSSTISILCSDGTF      643
LC_00401       VHSNGRLFYANPSFLLLTGYKNIGEIEKAGGLDTLLDAQKLPGSKRPLGSVSLYHANGTS      430
LAm_00040      VHSNGNLLYANPSFLLLTGYKNIEEIEKSGGIDNLLNAQKLPNSKRTLGSISLYHIDGTS      430
LPsy_00383     VCSKERLLYASPSFLLLAGYKSVEDIEKVGGVSMLLDAQKLSNG-NTHGSITLHRSDGTN      385
LAf_00635      VYSYGNLFYANSSFLLLTKYKSIADIEKSGGLSTLLNAQKLSKN-NTAGSVMLCRSDGTR      388
LAs_00286      VYSHGNLFYANPSFLLLTKYKSVDDIEIAGGLSTLLDAPKLSDN-NAIKPVMLYRSDRTC      393
               : *  .: *:. :**:*: **.  :**  **:. **:  :   . .    : :   : *

LCres_00213    IPVSVRLHSILWEGQKLLSLTVVPLIEEYEYHEP---NNIAPKYEKRIKAYIHALETEIT      700
LC_00401       IAISAHLHSIRWNGENSLAITFVPFERKNKPSNYSYSNGI-GHK--EEKTNSHNTEIETM      487
LAm_00040      ITVTSRLHSIRWNGEKSLAITFVHIDRNNKIYRPIDSSRIDNGK--KEKCNLSNTEIEAM      488
LPsy_00383     IAVSAHLHTIQWNRENSLAMTFVPFEKNNKLSDHISQNEIKSGY-NNTRTDANKMEIEIM      444
LAf_00635      IAVSAHLHTIQWNGENSLAMTFVPLEINNTFSENVSHEET-------NNPNMHKMEIEAV      441
LAs_00286      IAASARLHTIQWNRENSLAMTFIPFEKANQFPENMPQNGIEPED-VDTRINKRKMEIEVM      452
               *  : :**:* *: :: *::*.: :             .          .       * *

LCres_00213    QLNSILETASNGIVIISPDRKIQSINHPATLLFNYTSEEINGKSFTILFSSESQKIITDY      760
LC_00401       QLFSILEVASDGIAIINRDGEIISTNQSISKLFGYPSQDLLEQQFTKFFIREDQSMMREY      547
LAm_00040      QLFSILELASDGVAIINGDGKIISANQAISKLFGYSSKDLLEQKISNFFVREYQNTTNSY      548
LPsy_00383     QLCSILESTSDGIAIVNRDGTILSTNRAIGTLFDYPAQDIIGKPFKIFFTHDNQIIMNHC      504
LAf_00635      QLFSILEATSDGVAIINEDGLIISTNRKISKLFDYPTEDMLKKPFTMFFENNTQNVMHHY      501
LAs_00286      QLCSILEATSDGIAIINREGIILSTNRAVSKLFGYPVEDILRKPFTVFLEQNTPSVMNHY      512
               ** **** :*:*:.*:. :  * * *:    **.*  :::  : :. ::  :

LCres_00213    LDDISTDNAFHLLNQWKEVFGRKANGDSIPIFLTIKKLLSSSGYYATLQDVVQLKHNEEA      820
LC_00401       LSEILEFNPENKLE--KTTIGRTKEGELISLRVTIIKLPFSTCCCIIRDISEWKQEKKK 605
LAm_00040      ISEILKLKAGDKIE--KTTIGRTKKGELVSLRITITKLPFSIYYCMIIHDISEWKQEKKK      606
LPsy_00383     MAEISSIDLGKTLE--KTAVGCTREGKLISLRITMKKLPFSACYCLILHDISEWKQEKNE      562
LAf_00635      LVDILSLNQEQILE--KTTIGRTREGNILSIRIIIKKLPFSNCYSLIIHDISE----END      555
LAs_00286      LTEILSLDLRQTLE--KITLGSTKEEKLLSLRIIIKKLPFSSCYSLTMHDISEWKQEKNK      570
               : :*   .  . ::  * ..* . : . : : : : ** *     ::*: :    ::

LCres_00213    V----------------------------------------------------------- 821
LC_00401       LYDAKKIAEKENSHKSDFLARISHEIRTPLTAIIGFAEVIQNQKLGPVGSPRYIEYANYI      665
LAm_00040      LYDAKKTAEKENSYKSDFLARISHEIRTPLTAIIGFAEIIKNQKLGPVGSNRYIEYADYI      666
LPsy_00383     LYHAKKMAEKENSHKNDFLARVSHEIRTPLTAIIGFSEVIKNQRFGPVGSPRYIEYANYI      622
LAf_00635      LSHAIKKAKTESLHKSDFLARVSHEIRTPLTAIIGFSEVIKNQKFGPLGSPRYIEYANYI      615
LAs_00286      LSHAKKIAEKESSHKSDFLARVSHEIRTPLTAIIGFSEVIKNQRFGPLGNPRYIEYANYI      630
               :

LCres_00213    ------------------------------------------------------------ 821
LC_00401       NQSGNLVLDIVNDLLDISKIEAGQMNLHFESVSLNETISEAISLIQLYANEKRILVRTSF      725
LAm_00040      NKSGNLVLDIVNDLLDISKIEAGQMDFHFESVSLNETISEAISLINLYANEKRILIRTSF      726
LPsy_00383     DRSGNLVLDIVNDLLDISKIESGKMNFHFESVSLNETISEAISLMELYANEKRILVRTSF      682
LAf_00635      DRSGNLVLDIVNDLLDISKIESGKMNLHFESVSLNETVSEIISLVQLYANEKRILIRTSF      675
LAs_00286      DRSGNLVLDIVNDLLDISKIESGKMNLHFEPVSLDEAVSEAISLVQLYANEKRILIRTSF      690


LCres_00213    ------------------------------------------------------------ 821
LC_00401       SRVIPLILADVRSIKQIALNILSNAINFTPSGGQIIISTAYIKNKGVIFRVRDTGIGMNG      785
LAm_00040      SSEIPRIFADMRSIKQIALNILSNAINFTPSGGQIIISTAYIRNKGVILRIKDTGIGMNS      786
LPsy_00383     SNDIPPIFADLRSIKQIALNILSNAIHFTPSGGQIVISTAYTRNKEVVLRVKDTGIGMTD      742
LAf_00635      SDNIPQIFADLRSVKQIALNILSNAIRFTPSGGQIIISTTYTRKEEVVLRVRDTGVGMSN      735
LAs_00286      ANNIPRILADLRSVKQIALNILSNAIHFTPSGGQIIISTTHTSNEEVILRVRDTGVGMTN      750


LCres_00213    ------------------------------------------------------------ 821
LC_00401       CELEKAMQPFGQVFNSANVHREGTGLGLPLTKAMVEANMGKFSVLSTPSKGTLIEIIFPV      845
LAm_00040      YELEKAMQPFRQIINPTRVRKEGTGLGLPLTKAMVDANMGKFSIFSTPSKGTFIEIIFPV      846
LPsy_00383     YELEKAMKPFGQIPNSQQVRGEGTGLGLPLTKAMVDANMGKFSVFSTPSKGTLIEIIFTP      802
LAf_00635      YELEQALEPFGQIPNSQQIRGEGTGLGLPLAKAMVDANMGKFYVSSIPTKGTLIEIIFLP      795
LAs_00286      YELEKAMKPFGQIPNSQQIRGEGTGLGLPLAKAMVDANMGKFYIFSTPAKGTLIEIIFPL      810


LCres_00213    ----------   821
LC_00401       NMHHKD----   851
LAm_00040      NTHNKVL---   853
LPsy_00383     QKTA------   806
LAf_00635      HKLYDRN---   802
LAs_00286      YDTSHPHDCI   820
```

CLUSTAL O(1.2.4) multiple sequence alignment

hypothetical protein, COG5462

```
LCres_00752    MLKTSDFLIISVMIATAFITYSIKHRSELKREEVVRLEEAIKLENDTIDLLKVDWALLTQ      60
LC_00420       ------------------------------------MEHKIAEEQNYVELLKAQLAVLVQ 24
LAm_00466      MLKTVDLVIFALVIISITATYYIKHQSDLKTDHLKVLKHKIVEEQHYIELLKAQLALLVQ      60
LAf_01082      MFKTFDLIILGIILASISTTYSIKHKVENKKEELRILENKIVLEQNYIDLLKAQWALLVQ      60
LAs_01004      MFKTLDFIILGVVLASITITYSIKHETEGKKEKLRILENKITSEQNYIDLLKAQWALLIQ      60
LPsy_00654     MLKNFDLFMIVIVLVSITMTYSIKQQTENKRELLRSLESKILLEQDYIDLLKAQWALLVQ      60
                                    ::  *  *:. ::*** .: *:* *
```

```
LCres_00752     PSRLSTLGVFYKKELQLQPTDPLALALPVELPMLRSELPLDNRLIASQMNKPKIDLSQQK      120
LC_00420        PSRIKSLVSFYQKELRLHPTNTLQLISVDDLAKLKWRDFSENEKVLFP-RIHQIK--KYK      81
LAm_00466       PQRIKSLVSFYQKELQLYQTDTLQLISIDDLSKIKFRDFSRNAKVFSS-DTSGMR--EDK      117
LAf_01082       PDRIKDLVALYQKELQLQPTIISNLIAYDDLAHLKKKQSFSENRSDFSLKKVKIR--KYY      118
LAs_01004       PDRIKDLVSLYQKELQLQATNPINLITYDDLARLKKHTLLPENRSNLPKRTVERR--QHR      118
LPsy_00654      PDHIKDLVIFYQKELQLQPTNPVNLISYDDLSKLKRRFFLNENRFNLPKNKLKNV--PYQ      118
                *:::. *  :*:***:*  *    *    :*  :: .    :

LCres_00752     KFIKNKKLVFDNKGFDNLVTGAVQH      145
LC_00420        NQKKSR-------------------      87
LAm_00466       NSKEP--------------------      122
LAf_01082       QKVVQKK------------------      125
LAs_01004       KEIVQQQ------------------      125
LPsy_00654      KKIIHK-------------------      124
                :
```

CLUSTAL O(1.2.4) multiple sequence alignment

Flp pilus assembly protein, secretin CpaC

```
LCres_01313     MNSLLRQIFNSIRIFFISAFYILGIFFAQADNFYNNNSVIRVRLSEIGRDKNISVGLYKA      60
LC_00509        ---------------------------------------------MGRSKKITVGLDKV      14
LAm_00225       MNILQRIFVVFFMLIPSSLLSKPL---SLNSSVVEKPTVIKIEKSELGTNKRITVGLDKV      57
LAf_00403       MKYFLRTLFTILSIFLFSFDSLLA---VLPPIKDGNNTVINISYADIGKSKRLNVGLNKV      57
LAs_00478       MRYLQRTFFTMMSIFLFSSNPSVA---KLPPIKEANAAVINISDVEIGKGKKISIGLNKV      57
LPsy_00139      MKNFQKTLFMLFVIFISSSNFLLA---QSKLVDAKNASTIHIGDSAIGTSKKINIGLGKV      57
                                                 :*  .*.:.:** *.

LCres_01313     LVITFPDDIVDVLVSDPKKADVVTRSSRMIYLFGKEVGQASVFVFGANGKELLNMNVKIE      120
LC_00509        IILEMPSEVNDILVSDTVKMEVTLPSVNTVYIFGRKIGHSNIVILGRDGKELLNIEVNIE      74
LAm_00225       LILEMPLDVSDVLVSDPNKMSVKLYSVNTVYIFGQKLGRANIIIVGKDKKDLLNIEVNIE      117
LAf_00403       VILRVPSVVQDVLVSDPSKADVVMHSSNTMYLFGKDIGQANVILLGHDGKQILNLDITVE      117
LAs_00478       IILQVPVDVQDVLVSDPTKADVVVHSPRTMYLFGKNVGQANVILIGHDGKQMLNLDILIE      117
LPsy_00139      VILHLPSRVQDVLVSDPTKADVVVHSSKTVYIFGKSVGQANVILIGHDEKQLLNIDIFIE      117
                ::: .*  : *:****  * .*   *  . :*:**: .:*:. :.:.. * : *::**::: :*

LCres_01313     RDIGNLEANLRRFIPDSNISTEIVSDNIVLHGTVRTIQDSQKAVDLANAFIKGGEATTQT      180
LC_00509        RDFSTLQNNLRRFIANSNISVEMISDNIVLNGTVKSAQDSQRAVDLARIF----------  124
LAm_00225       RDAYTLQNNLRRFIPNSNISVEMISDNIVLNGEVKSAQDSKHAVDLATAF----------      167
LAf_00403       HDVVYLEATLRRFIPDSKINVEMVSGSVVLHGVVRNIQDSQRAVELAETYFAKNHRSSVN      177
LAs_00478       RDIAHLEMTLRRFIADSNIRVEMVSDTVVLHGMVRTIQDSQRAVELSETFLSQSGRNQ--      175
LPsy_00139      RDIRNLEMTFRRFLSGSNIHVEMLSDNLVLHGEVRTIQDSQHAVELSNMFLSNERNNL--      175
                :*   *: .:***: .*.* *.:* ..**.* *:  ***::**:*: :

LCres_01313     ASAGQSLSLQEDRRVSTVVNLLDIEGEDQVTLKITVAEVRREILKQIGFTNSLTRSASSL      240
LC_00509        -------------SDKKVLNLLNVECSDQVTLKVTIAEVSREVLKQVGFNHSIQAGG---     168
LAm_00225       -------------SDKKVLNMLKVECSDQVTLKVTIAEVSREVLKQVGFSHSIQEGSSAV 214
LAf_00403       -------NIGGNHNRSQVINLLNISGEDQVTLKVTIAEVRREILKQIGFQYNIMRSAT-P  229
LAs_00478       ---------YANSSSKKVMNLLNIAGEDQVTLKVTIAEVRRDILKQIGFQHSITGSSSGP 226
LPsy_00139      ---------YKTASGSKVINLLNIGAEDQVTLKVTIAEVRRDVLKQIGFQHTIS--SGGP  224
                          . *:*:*:.  .******:*:*** *::***:**  .:   .

LCres_01313     GHKLSSFSALGLTEGSSGQQETLINIGGVAGHYSLESALRALEQARVIRTLAEPTLTAVS      300
LC_00509        ---L----DISILGGGNGGDSSSLSIGTSNKYFTLKTFLRALEHARSLHTIAEPTLTAIS  221
LAm_00225       SSPL----NFNILSGGSAGQDNSLYIGTSNKYFTLKTFLRALEHASSLHTIAEPTLTAVS      270
LAf_00403       GSGK------GGAFFDQAG-FSNFSVAGFLDKFAFEGVLRSLERANAIRTLAEPTLTAIS      282
LAs_00478       SKSFA--ADFGGKFVSE---GGDFSVKGVLDRFSFETVLHALERATAIRTLAEPTLTAIS      281
LPsy_00139      SKGKR--IDFDGSLGGQ---GADFAMTTILDRFTFKSVLNALERATAIRTLAEPTLTAIS      279
                       .   ..      : :      ::::  *.:**:*  ::*:*******:*

LCres_01313     GQSASFRSGGERLYPTVDK-NGTTNFQTREFGVLLNFTPTVLAPGRISLRIQTEVSEPVT      359
LC_00509        GQNASFTSGGERFYRISDG-KGSYILKPYKYGIKLNFTPTVLSPGRIGLRISAEISEPSL      280
LAm_00225       GQSASFSSGGERFMRSFDS-NGKPSLQSHKYGIELDFTPTVLSPGRIGLRIKASISEPIV      329
LAf_00403       GQSASFRSGGERLYQSQDR-NGVANLSSHKYGVSLTFTPTVLSPGRIGLRIQTEVSEPSM      341
LAs_00478       GQSASFTSGGQHLYKTVSSSTGATSVTTHDYGVVLHFTPTVLSPGRIGLRIQTEVSEPVI      341
LPsy_00139      GQNATFRSGGTRLYRSVGA-NGTSTITPHDYGVVLTFTPTVLSPGRIGLRIETEVSEPVL      338
                **.*:* *** ::    .  .*    .   ..*: * ******.****.***. ..:.***

LCres_01313     PNAIATASTPPEFRTRRTETTVELPSGGSIALAGLIKSETQHGTLGVPVLSQIPILGALF      419
LC_00509        -----SAGSEPEYTVRTADTSVELPSGGTIVLAGLLKNSENYDSKSVPLLSQIPVLGSLF      335
LAm_00225       -----NS-GEPEYTMRTTETSVELPSGGTIVLAGLLKNAESYDSNAVPLLSQIPILGALF      383
LAf_00403       SI---GAGKVPEFRLRKAETTVELPSGGTIVLAGLLKDDIQQTRQGVPLLSKIPILGALF      398
LAs_00478       GV---NAGDMPSYRVRKADTTVELPSGGTIVLAGLLKDDIQQLKEGIPLLSKIPILGALF      398
LPsy_00139      GV---STTGEPEYRMRKADTTVELPSGGTIVLAGLLKDDIQQKRGGVPLLSKIPILGALF      395
                    :   *.:  * ::*:*******.*.****:*      ..:*:**:**:**:**

LCres_01313     RNKSLDRNETETVIIATPYLVKPVSRNSLSRPDDNFTLESDPTSFFLNRVHKVYRSGMDI      479
LC_00509        KNMSINNTATELFIAATPFLVKPVGVNDLVRPDDNLSLASDVKSLFLNRVSKIYGDKDAA      395
LAm_00225       KNMSVNRNATELFIAATPFLVKSVGEGDLVRPDYNFELSSDADNLFLNRVNKVYGSKDYT      443
LAf_00403       RQSDFVREEAEIFISATPFLVRPVAMNSLDRPDDHYAVENDSKSFFLNRVNKIYGTREE-      457
LAs_00478       RNSRFNREETEIFIAATPFLVKPVAMRDLSRPDDHYSVEDDAKAFFFNRVNKIYGPKEAS      458
LPsy_00139      RSNSFSREETEIFISATPFLVKPVAMNELSRPDDNYDIENDAKAFLFNRVNKIYGPKEAA      455
                :.  . .  :*.* .* ***:**:*. * .. * *** :  : .*   :::*** *:*

LCres_01313     PKAQRPYQGTIGFIYK      495
LC_00509        QNAEKGYKGAIGFIYK      411
LAm_00225       KNAEKSYKGAIGFIYK      459
LAf_00403       -IESNNYKGAIGFIYK      472
LAs_00478       EVEGQNYKGAIGFIYK      474
LPsy_00139      QGNGQNYKGAIGFIYK      471
                     . *:*:******
```

CLUSTAL O(1.2.4) multiple sequence alignment

Ribosomal protein L10

```
LCres_00877     MKRQEKQKEVMELSKIFNAYGSVVVAHYKGINVAQIKDLRAKMRMAGGCVKVSKNRLVKI      60
LPsy_00984      MNNQEKSVEISELSDIFSSSSSIVVADYKGLNVAQMKDLRARLREAGGGVRIAKNTLVKI      60
LAf_00865       MNRQEKSVEISELSKIFSSSGSVVVAHYKGISVAQIKDLRKKVREAGGGVKVAKNRLVKI      60
LAs_00017       MNRQGKSVEISELSKIFSSSGSIVVAHYKGISVAQIKDLRKKMREAGGGVKVAKNRLVKI      60
LC_00601        -------------------------------------MSRKKIRHALKFGILDRNLNKTV      23
LAm_00455       MKRQEKILEVAELGKIFSSYNSVVVAHYKGINVAQIGSLRKKMKEAGGFAKVVKNRLVKV      60
                                      * ::: *     : :*    .:

LCres_00877     AISDTSA-KGISGLFKGQSLIIYSSDPVAAPKVAVRFAKDNEKFVVLGGSIGENVLDATS      119
LPsy_00984      AIRDLGFVVDFADLFVGQSLIVYSVDPVIASKISVGFANDNSQFEILGGILEKDMLTQDS      120
LAf_00865       AVSDTSL-KGVSDLFVGQSLIVYSVDPIVAPKISVSFANDNKQFVVLGGILEKDILDQDS      119
LAs_00017       AIRDTSI-RGISDLFVGQSLIVYSDSPVIAPKISVSFSNDNNEFRVLGGVVEKGVLNQDS      119
LC_00601        FYNLSKP-SCFFYFFSGQSLIVCSKDPFVAPKISVDFAKSNNQFMIIGGVLDKGVLDKDS      82
LAm_00455       AIKDTDF-QGMSDFFSGQSLIVCSKDPVAAPQISVDFAKENDQFKIIGGILDKGILDNDA      119
                 . :* *****: * .*. * :::* *::.*.:* ::** : .:.*    :
```

```
LCres_00877   IEEMAMLPSLDELRAKILSAIQMNATKLVRIIKAPSSQVVRVISAYANKSE--   170
LPsy_00984    IKKIASLPNIEEIRSGILSAIQSTATRLVMLLETPQNHIVRVLSAFEEKNRQD   173
LAf_00865     IKRIASLPNIDGIRSMIISAIQFNSTRLVNLLNAPQTKIVRAISAFVDKNQQS   172
LAs_00017     IKQIASLPDLEGIRAGIISAIQSNATRLVRLLGTPQTQVVRAISAFVDKNQQG   172
LC_00601      IKEIASLPSIDVLRARILGCIQHNSIRLVRAISSPQARVFRVLSSLSEENRQD   135
LAm_00455     IKEIASLPSIDVLRARILGCIQHNSVRLLRTLNAPHARFFHIISACMEENKQD   172
              *:.:* **.:: :*: *:..** .: :*:  : :*  :..: :*:  ::..
```

CLUSTAL O(1.2.4) multiple sequence alignment

preprotein translocase subunit SecG

```
LCres_00494   MQIFVIIVHFVVVIALVGVVLIQRSEGGILGMGDG-SRFLSPRGAVDALTRITAILAGLF   59
LC_00685      MQMFVIVIHLLIVFGLVCVILIQSSDSTAFGSSSS-SRFINRGNYNSSKVRFTSVLAFLF   59
LAm_00828     MEIFVIVMHLLIVFGLVCVILVQSSDSTAFGSSSP-SRFLSRGASNNFQVRITAVLAFLF   59
LPsy_00849    MQIFLMTAHLIIVIGLVCTILIQSSDSSAFGTS---SSFTSVRSIAYSLARFTSILAFLF 57
LAf_00070     MQIFLIVAHLIVVFILVSVILIQSSDSSAFGSSSSSSNFTSVRSSAHSLWRFTAIIAFFF   60
LAs_00072     MQIFLMVVHLIVVVGLVCVILIQSSDSSAFGSS---SNFTSVRSTAHSLGRFTAILAFFF   57
              *:.*::  *:::*. ** .:*:* *:.  :* .   * * .         *:*:::* :*

LCres_00494   FITSIVLGILLRYDYDAKD-IINR-----IPKNSEKNSG----ILDSL---SPPNSSHSP   106
LC_00685      FATSILLGVMSRYEAVKYRKNLQKAMVDSKAKIEDSIP----SVSDKKVDISKESSATHN   115
LAm_00828     FSTSILLGFISRYKYMQYTKSMHKDVVASKDKTVDYNPIKSSSVTDKKVAVRNEPSASNN   119
LPsy_00849    FSTSIALGITSRYHSIKTKEDLRQSLASATNNKINYNSGNSNPNIDSSVP-EKKKY---P   113
LAf_00070     FATSLALGITSRYFSLKNKEALNQSLIDLTKNQVVDNSSDANYHKQVVDNSSDANSHTKS   120
LAs_00072     FATSIALGMISRYTSTRYKDNMHRSLVDSIKDQGNDNFGDGSSPKLDS------------105
              * **: **.  **        :.:       .

LCres_00494   GKEGKAVSPLQNSSDIPVNP--------   126
LC_00685      SK------KRSYISNNPKNKSPTHTSTK   137
LAm_00828     SS------KKSSMSNSRKNESIPHNSKK   141
LPsy_00849    SK----KNSLPLSSHVR-----------  126
LAf_00070     SS----KSKKSLSSAVPPNNSSS--SKK   142
LAs_00072     ------VSAKSSSSVVAGKRSSS--RTK   125
                    *
```

CLUSTAL O(1.2.4) multiple sequence alignment

hypothetical protein

```
LCres_01204   MADFVAVIRRAVDSLPENTPEMRARLYERARGAVVRQLEAMKPRPSEDILSRQFDKLEKA   60
LC_00692      MVDLVSVIKRAIDSLPENTPEVRFQIYDRARIAVSRQLESMKPRPPQEILYRQLSKLENA   60
LAm_01025     MVDFVSVIKRAIDSLPENTPDMRFQVYDRARIVVSRQLEKMKPRPPQEILDRQLRKLENA   60
LPsy_00059    MVDFVLVIQRAVDNLSENTPEMRSHIYERARVAVSRQLESMNPRTPRDILERQLSKLEKA   60
LAf_00692     MVDFVLVIQRAVDNLSENTPEMRSHIYEHARHAVSRRLEAMTPRPPKEILERQLSKLEQA   60
LAs_00585     MVDFILVIQRAVDNLPENTPERRSHIYEHARNSVARRLESMKPRLPKEILERQFNKLEQA   60
              *.*:: **:**:* ****: * ::*::**  * *:** * **  .:** **: ***:*

LCres_01204   IFQVEQQQQYLQQYLQQYLQQYLQQYPPQQQ---QYPPQQQQYPPQQQQSTWEKIP   117
LC_00692      ILKVESKHHQTINTIDKKIELD--------PPKDKISLDLPNGG-SINPSNCILLASRLP   111
LAm_01025     IFQVESKQHKIISVTDQ-----------------KMSLDVPDNG-GINSSNCILLASRLP   102
LPsy_00059    ILQVERKNKKFPRALDKDRVLL------------------APKS-HVNSKKNVFLTSRLT   101
LAf_00692     ILQVEKNNKKCPSSLHENKGLV--------I-------SKNNND-DVAVKKNVFLKSRVR   104
LAs_00585     ILQVEKQNQKSLHTSKQDKE------------------SDIPKS-SVTSKENIFLEPRLR   101
              *::** :::      :                          .:      ::

LCres_01204   VIPGTMPRR-------RPLENLSVNRGVGKSSIDVSGLAAPFRLNESNSIVNSQPLTQQF   170
LC_00692      VSSFSQNIDGSSNTRNTRLDNQHA-QKNRTAIYNFITKSLSYRRRNIFS---FPSYSQHS   167
LAm_01025     VSSFSHNDDIIS-NRNTISDKQYA-GKNMSITYSFIARSIYNKFINIFR---FSSYSQHS   157
LPsy_00059    SISSILRNRK----RKRTVDIVST-KGNKKTT-----THRPYQLRDILH---LSSNVQQG   148
LAf_00692     SIPLGVNNT-----DEKTVTILSS-LARNNVGENVSEKNFSYRLRDILS---FPVDAQES 155
LAs_00585     SISSILRSNK----HKKLANILSV-QGKSRTNTNLSPKNFSCRLREILS---FSVNTQHE153
                                    : :          *.

LCres_01204   ETNNLVPENTTNRIFYRNSAPRIRNTWDKLKRSKVNTAIHFADTRTD--FPKGGFLESTK   228
LC_00692      Y---------SNKSVSIN---A-DYGQASLKKIGLEQFF--PIHSYVSVFMKKCLLNTE-   211
LAm_01025     Y---------NNKSIFIN---A-DYTQGVLKKIGLEQFS--CINNYLFIVIKKYFLSKE-   201
LPsy_00059    S---------NAQILSME---SIEYNKNRLRRDKLLRKFSVSSSRSIFFLLQSYFSNKI-   195
LAf_00692     Y---------DPSTFPVA---AIEHDKNRLRRARLLGKFSPP-SGSIFFSVHNYFLNKT-   201
LAs_00585     Y---------DSSVSPVA---AIEHDKSRLRRGKLAGIFSFP-TGSIFWSVHNYFFNKT-   199
                          *::  :             :  : ..

LCres_01204   AISRSTSGFF-LSLGSRILRYIIICF-SILCFCGIGYLLWMSRNSIREIIVWVIKDRNID   286
LC_00692      ---RIFLSFFSRLYENNILKYCVVSFIFLGAFIGISYSFSKSQGNTSVPAS-KSLD-NNV   266
LAm_01025     ---YGFLSFFFKLYDNNILKYSAISFIFLGCFIGISYSISKSQVNTIGYIN-KLLD-NNS 256
LPsy_00059    ---RVFISFYTALLEYHFFKQSVFLVVFLGMMMGLSYSFWQNKVSFSHILENKILNRDSD   252
LAf_00692     ---RRLLYYYSVLSERNFFRYFVFLIVLLGVTIGLSYSLRKNKVNIVSSLSIKTLNKDNV   258
LAs_00585     ---RGLLSFYSALSEHHLFKYFVFLIILLGMAIGVSYSIGKSKGSITHFLRRESLDGGNV   256
              ::      .:::   . .  :    *:.* :  .: .          : .

LCres_01204   DKKISSSESSGGVSKTKITKRLLPDGSEIDAGEAIILDQALSSEGTVSTGISEKADESHG   346
LC_00692      KNQLF----IHANNIPKITRRLLADGSEIDVGSSYASDVSSSAFNNVFIQNSSVGDKDVK   322
LAm_01025     KNKLS----VLTNTVPKITRRLLADGSEVDMDSSNASDLSNPSFNNVFVHNSLVDDKDVN   312
LPsy_00059    NKKVL----HALGSRPKITRRLLANGSEVDMGTAISSPINSSNTSNVFFKNHIDSNDQ--   306
LAf_00692     DKG-N----TGLNIRHKITRRLLEDGSEVDMGPSAVSVSNSSNISNILFKNHTDMD-K--   310
LAs_00585     DKK-N----VFSGIRPKITRRLLEDGSEVDVGPSTIPVADFANTSNIAFKNYIGGD-E--   308
              .:          ***:*** :***:* . :        ..:       : .

LCres_01204   NPVHSRLEDKSLSGNNANKTGDKIFLYEEGVNDGGQQVVVPGNVVWSIQKKKDEKGYSDP   406
LC_00692      NNFVHISKGNKSEKISGI-EGASIAFINK---GYGNPLAIFKNVAWSLEQ-----GSQGL 373
LAm_01025     NNVVSLSKGSKLEKTAGN-EGASIAFINK---GYGNPLAFAKNIAWSLQE-----GSQGL   363
LPsy_00059    -AVSHILERKKSGTENPI-DEDGRVFINQ---GSGRSSIFAGNIFWSLQKEKT-QGLKGL   360
LAf_00692     -SVVHPLEKKNREKENSL-IGESKVFINK---GKGRSSIVSGKILWSLQQEKS-QGLKGL   364
LAs_00585     -NSTFVLGKKEIEEGNPL-IGEGRVFINK---GRGQSSILSGKILWSLQQEKS-QGLKGL   362
               ..           : ::   . *.   .  :: **:::     * ..

LCres_01204   VVRGNISVPERGLFAMLTFKRNTDTSLPASHLIEIVFSLPKSFQGGGIESIQEVSMKNNE   466
LC_00692      MIKGDVPTIGSDLSASIFIKRNFDISLAATHLIEMEFSLGKENQGGDIADLRQISMRKTE   433
LAm_01025     MIKGNIPTIGSDLSALIVIKRNFDISLAATHLIEIVFSLEKGNQGGDITDLRQISMRKTE   423
LPsy_00059    VIKGDIPTINNAFSASITLKCNADIALSVTHLMEITFSFPKESQN-SIVDLRQISMRKTE   419
LAf_00692     VIKGDIPMIDNEFSASIILKCNADIALSVTHVMEIMFSFSPESKD-AIIDLRQISMRKTE   423
LAs_00585     VIKGDIPMIDNAFSASMTLKCNADISLSITHVMEIMFSFPKESQD-AVVDLRRISMRKTD   421
              :::*::      : * : :* * * :* :  :*:.*: **:    :.  : .::.:**::.:

LCres_01204   HGVSNHLMAVAAKITNDFHMIALNNSSEAQKANLNFLENFQWIEIKITYRNGKKITLVID   526
LC_00692      KGPSVFFDANIFRIAKNSYLISLKGS----LNNSEILSEYRWMDIPITYSNGRRIVLTID   489
LAm_01025     KGPGVFFDSNIFRTAKNSYLISLKNS----LNNSEVLSEYRWMDIPITYSNGKRIILTID   479
LPsy_00059    NSPSILIDSNIFRISKNSYLISLKGDAEDFLRNSKILEEYRWIDIPITYHSGQKITLTID   479
LAf_00692     NSPSIFMDYNIFMISKNSYLISLKDFEEDSFRNSKILEESRLIDIPITYRSGKKIILTID 483
LAs_00585     NSPSVLIDSNIFVISKNSYLISLKGSEEDPFRNSKILEEYRFIDIPITYRSGQKILFTID   481
```

```
                :. .  :       ::: ::*:*:.       * :.*.: : ::* *** .*::* :.**

LCres_01204      KNKDGTEAFKTAIKEWKEFDK----                  547
LC_00692         KSRAGAEIFKMAISNWEGQTKNNLI                  514
LAm_01025        KGRTGADIFKMAISNWESQTKKSIS                  504
LPsy_00059       KGKVGSDVFKSAVMDWKDHSK----                  500
LAf_00692        KGKAGTEIFKSAIMQWENRSKQ---                  505
LAs_00585        KGKKGADIFKSAIMQWENRSNN---                  503
                *.: *.:: ** *: :*:   :
```

CLUSTAL O(1.2.4) multiple sequence alignment

pilus assembly protein, TadG/RcpC

```
LCres_01108      MCTRETNVSQLYFKGMQKLAKAIMLPYAFS-----NMDIKVYGYWTDDNKQTVQKWGWPN          115
LPsy_00013       MIAQETIINTKFLDSFNTFLDATMLPYRLQ-----NKTIAITGYWIDEKNNVKRMWYWPA          114
LAf_00735        MIAQESSIDKSYLEDFEKLLTSTMYPYIFN-----DCSIMITGYWVDSDKKVTKKWSWSS          114
LAs_00628        MVAQETSINKQYLQGFENFLRATMYPYRTP-----NHSIIVTGYWLDNKQIVRKMWNWSS          114
LC_00833         -----------------------MYPYHYKPNNNYNFSINIVGYWIDENMHVSKEWQWSS          37
LAm_00983        IVAQENSINNRYLDNFSLLAKATMYPYSYNSI-NHKISIKIVGYWIDDRSVIHRKWFWSS          118
                            * **        .  * : *** *.     : * *

LCres_01108      NAKD----PGPLPSVADPKVFVTVTTVISLQYKMLGFL----SNQWSEINIVKTDSLRQRE          167
LPsy_00013       DSG--SIKDDIPKSIMDPSTFIVRASVSTQYHMVLATPLLPFTM-SDINMNKVYYYRQRL          171
LAf_00735        PNIGITVKEDVPNSIKDYSSFVVRTEVTMKYQTFLMSRIFPNSLNGIIPLDKVFYYRQRL          174
LAs_00628        SNVKVE-REDIPASIKDASTFIVRAEVSINYRTLVFSKILPDSLKGDIVLRKVYYYRQRL          173
LC_00833         SNNP-NIACNIPEPMRDKSVFIVCSNVSSYYPYTFFSNDLLPADFLRMNINKVFYYRQRL          96
LAm_00983        DKSS-SSNCKIPDSMIDNSVFIVCSDVSGSYPYTIFPKDFSPSYIKDIKINRTFYYRQRL          177
                          : * . * * : ::  *                : : :.   ***

LCres_01108      GKEIICSDCSS-                    178
LPsy_00013       GDQIECKDCFKR                    183
LAf_00735        GGEVICRDCQT-                    185
LAs_00628        GDQIVCRDC---182
LC_00833         GDRINCVNCSKS                    108
LAm_00983        GEIITCYDCIN-188
                *  : * :*
```

CLUSTAL O(1.2.4) multiple sequence alignment

50S ribosomal protein L9

```
LCres_00357      LKEQALNLAQILDKKEFIVIRSAGEAGHLYGSVSARDIIDILNKENFIINRSQINLNTPI          120
LAf_00276        KKIQFEEMATILEKKDFFLIRSAGETGNLYGSVSSRDIADLLAEEGFQINRGQIDLRSPI          120
LAs_00205        KKAKYEGISKDLAKKNFSLIRAAGDTGYLYGSVSSRDIADLLIEEGFDVNRGQINLKSPI          120
LPsy_00489       QKKKFEEMAKVLAEDVFSIIRSAGDTGHLYGSVSSRDIADLLLEKGFNINRGKINLKVPI          120
LC_00973         ----------------------------------------MLTDEGFNINREQIRLKSTI          20
LAm_00345        IQSKFEELSKVLEGKSLTIIRSAGDSGQLYGSVSTRDIASLLNEEGFKINRDQIRLKSPI          120
                                      :* .:.* :** :* *.  *

LCres_00357      KSIGLHEVTISLHPEVDILIKLNVSRSEEEAKYQATGKSFASPESYYVFDQESLEEENLS          180
LAf_00276        KSIGTHNITILLHADVSTKITVSIARSSEEIIQKEKTLEKAEADTKAEAESE--AEE-VN          177
LAs_00205        KSVGIHNIMISLHADVSTTITLNVARSTEEMVKQDILGEHEEEILS--------------          166
LPsy_00489       KLIGVHNIEILLHADVTATITLNIARSTEEMVKQEKTLKPEEEITEITS-----------          169
LC_00973         KSIGIYDVTILLHSDVETAIKLNVARSNEEAIIQAKSAETKDEISDQPI-----------          69
LAm_00345        KSVGMYNATISLHSDVEAKINLNIARSNEDAKIQEKSVKNQDKISDQST----------- 169
                * :* ::  * ** :*   *.:::** *:   :    .  .

LCres_00357      DFENEIEEKPE                     191
LAf_00276        S----------           178
LAs_00205        -----------           166
LPsy_00489       -----------           169
LC_00973         -----------           69
LAm_00345        -----------           169
```

CLUSTAL O(1.2.4) multiple sequence alignment

pilus assembly protein, N-terminal domain-containing, CpaI

```
LAf_00411        MANIRTTLALSIFIFIVARIYPAFPQDQIQKAPPPIPTPNYPIP--DKKIIKQKPPLL--   56
LAs_00468        MA--RTQIALALSFFMITHSYYAFSQDEIKKNNPTLEK-KPIVL--MKHEIQEKKPL--- 52
LPsy_00146       MA--RIQIALVLSTIMIAYSYPAFSNDETNKNDPSANRQRPILP--LKHAKEEKKPILKP          56
LC_00985         ------MSSIVLSTFMLVDINHEFLIANYSYASTIYEESKETKPQLSRNINQAKPSNLKP          54
LAm_00217        MK--NRPITIVLSTFMVAYINTGLLNSNHIYANSNSKED-AIKPKI--NTEDVNKSKLNT          55
LCres_01324      ------------------------------------------------------------   0


LAf_00411        ------------PTHRNHQEAIKHANPKTKVRRIPPPHSPINKTLPTKPHEKEEAKNLRV   104
LAs_00468        --------PPLPPLHRKRQREIKNETVRK-------KGIP---TTASQKTPEDNSNRIRI   94
LPsy_00146       LQHEKKGAKPTPPLKHARPEKRRTEIQKN-------ATKPRTTNTPSPQPTTNDEKRIRI          109
LC_00985         QNVRPNDTIS--KQKSHINSHIKSPNSKTNINNTKKITGTTSNTKNV-NKTIEDKKPLRL          111
LAm_00217        QNLNHSNKAS--AKKGHDNSRIKGHNSRIK--GRPNININNAKKIDL-DETIKEEKYLEL          110
LCres_01324      ----------------------------MKFIMTIIIFA-TYIIFYINPALAENKNQIHI   31
                                                      .: : :.:

LAf_00411        AIGQSLILGFNTKITKVVVGNEKVIDVLALETENSVVVTGKEIGITNLIVLGKDNKVILD          164
LAs_00468        AVGQSLILQFDVLPKQVIVGDDKIVDVLALEKEKTVVITGKNLGSTNIIVLGHNNDILLD          154
LPsy_00146       AIGQSIVLKFDSLPSEVIIGNDQITDVLALEKEKSIVITGKKSGLTNIIVLGAGNKVLLE          169
LC_00985         EMGKSLILQFSTPPTQVIIGDALIANIIALEKEKRVIVTALNYGSTNLVVFGEDGNTLLD          171
LAm_00217        EMGKSTILQFTEIPKQVIIGDATIANPLPLENEKRLILTPLRSGSTNLVVFSKDGNILFD          170
LCres_01324      PMGESKIIKFDMPISKVIIGDVRVLDAIETDP-QTIVLTTKNYGSTNLVILDKEGNIVID          90
                :*:* :: *    .:*::*:  : : :  :  : :::*  . * **::::.  .. :::

LAf_00411        IEATTFANEKNTVRVYSPGNKAFLSCTPRCLPYTNTWNDIMTK------               207
LAs_00468        TEIATFANEQSTVRVYTPGTLSFLSCTPRCLPSSPPVR----------- 192
LPsy_00146       TDILSFPNEHDTVRVYTPEKLSFLSCTPRCLPNSENL------------  206
LC_00985         TEVITFKDDKNTVRVYKPDQINTLYCSPRCHLEKHKSSE----------210
LAm_00217        KTLITFINEKHVVTIYEPDKIKKLSCFPKCDLKKYNDYKGDNQQSYSNS               219
LCres_01324      KTVIVTVSENNTVRIYRQTKREVFSCNPNCESNEIIPNTVK--------               131
                  .:: .* :*      : *.*
```

CLUSTAL O(1.2.4) multiple sequence alignment

hypothetical protein

```
LCres_01333      --------------------MLNIPKLFTRDNFNYCYMQGKNHSKRVKRLRIILPVMSVV          40
LC_00994         -------------------------------------------------------MITLV  5
LAm_00211        --------------------MINFDHYDQ-DRILLQRQKAYQNSMIVKFLKIALPIITII          39
LPsy_00154       --------------------MDDMNELDRREKLLLQRKKYNQHFKLIRFLKFFLPTLSVV          40
```

```
LAf_00418       MIWITEKGNRNGIKGIISMTMNDVDQLDRIGKISSQRREYRKHVKFIGFLNFFLPVITIF       60
LAs_00460       ----------------------MDHLDRRNKILLQRSKYRQHVRFIQFLKFFFPFATVT      37
                                                 ::

LCres_01333     FSIGILLLSIENTRFSLSIPLDLT---NIDNNKIMMKGPKISGYNNDGSHYTMHAGKAFY     97
LC_00994        LLAWLLFVSWI-RLHSISMLGDFNSTSPAIPGQMNMQKLVMLDYKKNKKVFSIVADSVKA     64
LAm_00211       ILSWLLFISWV-RFHSTSVLGDFDIIDNVIPRQINMQKLIMLDYKNDKKAFSIIADSVNA     98
LPsy_00154      LLSWLFFSSWV-RIKHFSKPA----ADFLNFEFMVINKFILSDYSRDKKRYSLSAKRAQD     95
LAf_00418       MICWLSFSSWE-YARNFSKPL----VDFLGFEPMVMKKFILSDYSENQVRYSLMAEHAKI     115
LAs_00460       IVGWFLLSSWI-RISTFSKSS----IDLLDFEPMIMKKFILSDYSKDRVKYSLVAERAKT     92
                :  : : *       *             : ::  : .*..:  ::: *  .

LCres_01333     DISN-PNYVIMKDIEASLPIKNT--NRAHLIAASANFDRVQDVLNITVPFTLNLENN-IR     153
LC_00994        NIDN-QNMLFLHDINLSIPLKAY-DRDINIVTNSVKFDLYNNMLDINEPFTMQIRDD-TK     121
LAm_00211       NINN-QNMLFLHGMNLNIPIKAY-NGDINIIADTAKFDVYNNLLDINQPFTMKIRDD-TK     155
LPsy_00154      NEGS-KNTILLQDLELVMPIP--GYDEMRLLANYASFDLYRNMLNIQHPFKIIFNNNDTQ     152
LAf_00418       GFNS-KNIVFLKNFELSIPIEKD--DNMMVVASSANLDLRNNVLDITQPFKIILKDN-VQ     171
LAs_00460       SFNSGKGIIFLQDFELTVPTQRSEYGDMYLFAHSARFNLANHTLYISQPFKMKVKDN-LR     151
                . ..  . :::.::  :*         ::: . ::  .. * *  **.: ..:: :

LCres_01333     INFNSATFYIKKTALETKSPLSVFSSYGSIFAQSFNTKDKGHVMIFSGNVKMLIEPRVLQ     213
LC_00994        IDYKSATIDVAKSTLISKEDLFFTSPKFRVYSKSFDMGSNGKTVIFSGGVFATIKR----     177
LAm_00211       IDYKSAVINVKKSTLVSNEDSVFTSPKFKIYSKSFNMGDDGKTVIFSGGVFAVIKR----     211
LPsy_00154      LYFKTAVMDIKKIVLNSSDAVKFTSSTFTLSAQSCSIENDQKRAIFSGDVSVVIHPKILH     212
LAf_00418       LDFKNAYLDINKLAISSASPVIVTHSDFVISAQSVKIDKNSRNAIFVGDVSVIMKPEILQ     231
LAs_00460       LDFETAVLDVKNITINSSDPVIITHSDFVLSANFARIENSSRSAVFAGQVSVVVNPGVLQ     211
                : ::.* : : : .: : .   .     : ::     .. :  :* * *   :.

LCres_01333     KEKNEKRILK  223
LC_00994        ----------  177
LAm_00211       ----------  211
LPsy_00154      KKV-------  215
LAf_00418       RKK-------  234
LAs_00460       KKEN------  215
```

CLUSTAL O(1.2.4) multiple sequence alignment

outer membrane protein assembly factor BamA

```
LCres_00081     MEKRK----LSKALLS-------NVVYASVLCLELIVSLSLYSNEASASVVKKIEVRGIR  49
LC_01015        -----------------MFYRLFNFIFFIVIGAALYVP---SAYGSSRLIVNSIKTQGVT  40
LAm_00186       -----------------MFYRLFRVFGFFVFIGAIMFVP---FAYGANKLIVNSIKIKGMT  40
LPsy_00232      MGKDKKDSYGGKEQLKQYFPIYLRMGFFLLFCSIISVS---AVHGANAFSVSKIEVQGAT     57
LAf_00495       MYRKTKYFCGFHKLLKRSFSRP-LVGFFVFSYVFFAVS---VVYGSDSFVVNDIKIRGAV     56
LAs_00430       MHKSTEDFRRIKRLLEKYFPRSFQMGFIILFYAIFGLS---AVYGSNTSIVRRIEIRGAT     57
                                        . :  .    : :       :.   *  *: :*

LCres_00081     RVSADSIRSNIVISPGKVFSGEEIDDSLRRLYRTGYFSDVKISVSGSVLIVFVKENDLIN     109
LC_01015        YRDDKLIISRIPIIPGKYFSDEDIDSSIKNLYSTGYFSDVKIDIVDSVLVINVVENKIIN 100
LAm_00186       YRDDKLIVSRIPIIAGKYFSDEDVDASIKYLYSTGYFSDVKIDVVGSVLVINVVENKIIN     100
LPsy_00232      DIGRKLVLSRIPIVVGKYFSDNDLDDSVKLIYSTGYFSDVKIKVIDSTLFIDLVENKIIN     117
LAf_00495       NVSKQIILSHIPITVGKSFSEEDLDSSVKNLYAIGYFSNVKINVVNSVLIINLVEKQIIN     116
LAs_00430       NVGKEVILSRIPVVVGQSISDADLDHAVKNIYAMGYFSNVKIKIVDSVLIIDLIERKIIN     117
                  . : *.* :  *: :*  ::* ::: :*  ****:***.: .*.*.: : *..:**

LCres_00081     QVVFNGNKNIKDDKLQLIVKARPTVAYSRVLVDSDISAIKKAYSAIGRSEVSVSSQVFSV     169
LC_01015        QLVFSGNTKIKDGDLERIIGIRSSSSYDKAVVDSDVNLIKKTYSSIGYPNTSVDVKMYQV     160
LAm_00186       QVVFSGNKKISDASLEQLIGIHPSSSYNKDLVDSSIKLIKKYYSYAGYPTASVDIQVYNI     160
LPsy_00232      GLVFIGNNNVKNTYLERLVRSRPTTAYDKGIVSSDVVRIKQAYASIGYLNVSVDVQANVV     177
LAf_00495       HLFLSGNDNLKDDNLRLLIHSRDSFGYDEYTVKDDVRIIKEAYASRGYLNVVVNVKKYSI     176
LAs_00430       HLFFSGNNNLKDDQLKMIVRSRSAAAYDEDTVNADVHNIKQAYASIGYLNVMVKVQHHSI     177
                :.: ** ::.:  *. ::  : : .*..  *. .:  **: *:  *   . *. :   :

LCres_00081     GEGRVNLAYVINEGGRTKLGKINFVGNKAYSAVRLRSAIISRQSNIISFI-TRSDIYSKE     228
LC_01015        SPGVINLGYVITEGIRTKISTISFLGNNNYSNNRLGRVISSKTSGYFPFLAMGTDIYSKE     220
LAm_00186       SPGTINICYVITEGIRTKISNISFSGNKSYSSNRLGRVISLKTSGYFPFLAMGADIYSKE     220
LPsy_00232      SPTMVSLVYTIQEGVKTKINTISFVGNKAYSNTRLERIISMKKSGYFSS--GGVDIYNED     235
LAf_00495       SPTRINLTYAIDEGFKTTIDSIRFKGNNSYSQARLKSVISLKTSGFFLF--GEEDVYSKE     234
LAs_00430       SPTTLNITYVIEEGVKAKINSIRFVGNKNYSHARLERVISIRTSGYFSF--GKTDVYSKE     235
                .  ::: *.* ** ::::..* * **: ** **  *  * : *. :      *:*.::

LCres_00081     KLDADGEAIRKFYSERGYADFQIVSSQAFFDQAKNSYSLTFNINEGARYKFGNISVQSSV     288
LC_01015        RLNDDEQLIRNFYYDRGYAGVK-VTSKTVLNN--NRYDLIFNIDEGLVYRIGSIVIRSNL     277
LAm_00186       RLSYDEQLIRNFYYNRGYAGVK-VSSKAVFNN--NRYDLTFNIDEGSVYRISKIVIVRSDL     277
LPsy_00232      KLRFDENAIRQFYYNRGYAGIK-VSSQAILDNKKNLYDLIFKIDEGEIYRVGNVTIRSSL     294
LAf_00495       RMSYDEESIRKFYYDRGYAAVK-VSSRAFFDKAKNSYSILFDIDEGRIYRVGNITIKSTL     293
LAs_00430       RMSFDEEAIRAFYHDRGYAAVK-VSSQVLFDKQKSGYVLIFQIDEGEIYTVGNISIQSTL     294
                ::  * : ** ** :**** .: *:*:..::: . * : *.*:** * ...: ::* :

LCres_00081     QDIDPDKLRKFIQTKTRDVYNFNKVEKSIEAMSGYLTSIGKPFIKITSRISRNLQTSAAN     348
LC_01015        QYVSYDKLLSLINNKSGDLYSAKVIEENVIRITNYLYSIGNPFVDVTSRINRDFINNTVA     337
LAm_00186       QSVPHDKLLSLINNESGDLYSAQVIEENVLKITNYLYSIGNPFAYVTHRVNRDFINNTVN     337
LPsy_00232      EEAKDEKLLRLIQAKSGAVYNPQWIEKTINNISKYFLSIGKSFVRMDSRINRDVAKGIVD     354
LAf_00495       QDFTKDMLLSLVQTRSGDVYDPRKIEESTENISKNLFFKGKYFVRTNSRINRDFAKGIVD     353
LAs_00430       QEIQKKTLLSLIRIRSGNLYNPQEIKESSEKISKYFFSGERPFVRVKTRINRDFAKRIVD     354
                :    . *  ::. .:  :*. . ::::   ::  : :   .*     *:.*:. .  .

LCres_00081     VEYLVDKGERIYLERIDIRGNVYSRDFIIRREFDLSEGDALNESMITTAKRRLMDTGYFT     408
LC_01015        VEYLVDQGRPVYVDRIDIKGNDSIYDYVIRRELGLSEGDPVNQVMIERAKRRIMATGHFS     397
LAm_00186       VEYIVDQGPPLYVDRIDIRGNVSIYDYVIRRELGFSEGDPINQAMIERSRRRIMATGHFS     397
LPsy_00232      VEYLIDQDSPVYIARIEIEGNDVSHDFVIRRELGFSEGDPINAMMIERAKRRIKNTGFFS     414
LAf_00495       IEYIINYDSPLYIERIEIEGNSQSHDSVIRRELDFSEGDPISLAMIERAKRRIMATGYFS     413
LAs_00430       IEYLIDQGSPLYVKRIEIEGNDQSYDSVIRRELELSEGDPINYSMIERAKRRIMATGYFS     414
                :**::: . :*: **:* **   * :****: :****: :**** :.  **  ::**:  **.*:

LCres_00081     SVNISKVLGS-APDRVILVVEVQEQSTGRAGIGTTYSGRGV-FGLEASVEEHNFFGRGQY     466
LC_01015        DVQIDLLSSV-IPDRVLLIVQVKQSSDSTITIAASCGDSLK-CSFDAGFDDGNFLGKGYN     455
LAm_00186       DVLIDQLSSG-IPDHVLLTIRVKQSSDAVIKIGASCGDSLE-CSFDSGFSDGNFLGRGYD     455
LPsy_00232      EVNVFQLPTD-VPDRVVLKIHVKQLDSTSIGISLNYGSNKNSSDLEVFFSDNNLFGRGYK     473
LAf_00495       KVNILKLPAN-VEDRVVLRVIVEQLSSGSIGLSTNYTVNEG-TGIEGFIADNNFFGRGYR     471
LAs_00430       EVNISQLPANDVSDYVILRVSVKQLSAGSVGIATNYEVDKG-MGVEGHIDDNNFFGQGYR     473
                .* :  :     * *:* : *::  .    :. .     ..:  . *::*:*

LCres_00081     IRIAAGAGDSKSRSYNLSFTEPYFLGHRIAAGFDLSAVQSSDNTFFSEREHGVDLRVTVP     526
LC_01015        LGLKLSFGRDQLKDFNLRLRDPYFFCSRIGAGFDSLKSYSID-QSLGRNKSGLMLQAHIP     514
LAm_00186       LGLNFSFSGNNFRDYNLNFVNPYFFNTPIASGFNLMKSYSTD-KFFKKDISGLRLHARFP     514
LPsy_00232      QSIALS-----LTNKAYSFENPYFLGSRIAAGFDLGQSKLTE-SFGNVDKQLHSAHVTLP     527
LAf_00495       ARISLGVGHYRLRNYVFSFENPYFLGSRISSGFDIRRSSFME-GFLATEGKYGSVYFTFP     530
LAs_00430       ARLAAGFGRHAVQNYTFSVEDPYFLGSPISAGFDLQKTHLED-GSLDINDESAAVRMIVP     532
                 :  .        .  .:***:   *.:**:      :                   .*

LCres_00081     ITEEIGNTFRYSYKHMNYKGIGD-WKNPANLSDPYRELIRVGKWNTSAVSQTIAYDTLDN     585
```

```
LC_01015        ILENISTTSKFGYNNLKYDKIPKSFADKADTDAMISELVSHGSFNSLSVVQSIQYNTLDN      574
LAm_00186       VLENISTTSKFGYDVLKYGNAD-----NNNSSDFNRELVSHGKFNSLFVSQSVEYSTLDN      569
LPsy_00232      ITETISATSSYSYRSLQYQSAPG-----KEVSSVYKSLINGKKFITHAISQHFVYSTLDN      582
LAf_00495       ITEKISTTSSYNYKALKYSSSSM-----GEIHEIEKMLVNHGEFSGHSISQDIVYNTLDS      585
LAs_00430       ITESISTSFKYDLRFLQYGAISE----KEKIPSIYTTLIEHGKFSSHSISQSIIYNTLDN 588
                : * *. :  :.   ::*           .       *:   .:    : * . *.***.

LCres_00081     HLLPREGILAKFTHEYAGLGGDSKYYKVLGKVSYFQLLIDDADVIGSVSAGAGHVMPING      645
LC_01015        MKMPRSGLLVNASYEYAGFGGDSDYHKLSQRTDYFNLLSDNYDIKPIK------------622
LAm_00186       INMPRNGLSLSSDYEYAGFRGDSNYHKLTQMAHYFKLLSDNYDIIGSMRFRMGHIFPRNE      629
LPsy_00232      QIMPREGLMMVSSYDYGGFGGDSRYHRIGFKSSLFHLLSDNLDIIGSLKLGYGYIIPTNK      642
LAf_00495       QVIPREGIFMKSMYDYAGLRGDSKYHLIKYGASYFNLLSDNYDIVGSLKFGYSRVIPVDK      645
LAs_00430       PIVPRKGMLISSSYDYAGFGGDSQYHRIGSRASYFYLLSDDSDIVGSLRFGYGCVIPSNK      648
                 :**.*:     ::*.*: *** *: :      * ** *: *:

LCres_00081     KLYIFDQFMFNPE-ELRGFKNKGIGPRMAN-GDPVGGNTYFTAGAESNFPLPLLPRDLNL      703
LC_01015        ------------------------------------------------------------      622
LAm_00186       NLQFFDQFMIGPG-NLRGFARSGIGPRLKKDGLAIGGRTYVSASAEVDFPMAFASDDSGL      688
LPsy_00232      YLQIFDQFSIDTD-DLRGFADKGIGPRVD--GDAIGGKISLSASAAISFPMPFIPPSIGL      699
LAf_00495       NLQVFDQLSTSSD-VLRGFADRGIGPRVN--GYAIGGNTSFSASASVSFPMPIINPQVGL      702
LAs_00430       NLQLFDQFSVSSNYYLRGFAYKGIGPRVDK-KYAIGGKIYSSASAAVSFPMPLVPERAGL      707


LCres_00081     RGSFFVNAGTLYGNSVDIGS-SGPLQGDSPSFRVSAGVGLMWHSP-IGDIGVYYAVPLHK      761
LC_01015        ------------------------------------------------------------      622
LAm_00186       RGTFFVDTATLHNNAFRLNN-NSDIDGNDPYFRVAAGVSIVMHVPIFGKMSIYYGIPILK      747
LPsy_00232      RGSFFVDSANLYGNNFPWRTVGGNLEGSEGFLRISTGIGISWDLS-YVTAGVYYGIPLRK      758
LAf_00495       RGVFFVDSATLYGNDFHLDISKNKLEGNDSFWRVATGAEIAWDSP-IGAISLYYGIPLRK      761
LAs_00430       RGAFFVDSATLYANHV--ALGADKLEGNDSFWRVSTGVEIMWNSP-LGMMGVYYGIPLRH      764


LCres_00081     EPYDRLRRISFSLGNRF-      778
LC_01015        ------------------      622
LAm_00186       QSYDVVMPFGFTIGNSI-      764
LPsy_00232      KSYDKSMRIGFYIGNAAR      776
LAf_00495       QSYDTTMRWGIQIGNRFR      779
LAs_00430       REGDKIQQFGFRIGNRM-      781
```

CLUSTAL O(1.2.4) multiple sequence alignment

RIP metalloprotease RseP

```
LCres_00080     -MSFDLSYVIPFLSVIYLVVLVHEFGHYIAGRLCGIQAVVFSLGFGPEIFGFMSRSGVRW      59
LAf_00496       --MFGLDYLLLYIGSISIIVFAHEFGHYIVARLCNIRVMSFSIGFGPEIFGITDRSGTRW      58
LAs_00429       --MFWLDCFLLYTVSLIIIVVIHEFGHYMVARLCNIRVLSFSVGFGPELIGITSRSGVRW      58
LPsy_00233      -MMFLLDYLLLYFGSIFIIVFIHEFGHYIVARLCNVRVISFSIGFGAELIGFTSRSGTRW      59
LC_01016        ------------------------------------------------------------      0
LAm_00185       MIPVFLEYFFPYVLAICFIITVHEFGHYIAARLCGVKVRVFSIGFGPELYGITSSLGTRW      60


LCres_00080     KFSAIPLGGYVRFLGGERSSHVQSASSVVKNSKDKGQNSFLGASLWKRSLVVVAGPLANF      119
LAf_00496       KISYIPLGGYVSFSEDE-----------------KDPRSFVCSALWKKSVTVLAGPFINY      101
LAs_00429       KVSLIPLGGYVSFSEDE-----------------KDMRSFFCAAPWKKILTVLAGPLANC      101
LPsy_00233      KVSAVPLGGYVRFSEDD-----------------QDVRSFVCAASWKKILIILAGPFANC      102
LC_01016        ------MGGYVGFIEDE-----------------ADKNAFASVNAWKKILISLAGPLANG      37
LAm_00185       KISLIPLGGYVSFVESK-----------------DDNHSFKNVNAWKKMMISLAGPFANW      103
                 :**** *  ..                  . .:*     **: :  :***: *

LCres_00080     LMTAMIFTFLFYKNGIIIIDPIVSQIEPGSPAAEAGIKPGDLLVSVDGHQISSFQDVMLY      179
LAf_00496       VMAILFFAFFFYNTA--VIDPVVFKVFPGTPASIFGIKVKDRIVSLDGTAVSTSEDVAFY      159
LAs_00429       VMAILFFTFFFYNTG--VMKPVVSNVSPASPAAIAGVKKGDCIISLDGITVSAFEEVAPY      159
LPsy_00233      IMAILISTFFFYKTG--MIESVIFDVYPNSPAAISGVKAGDRIVSLDEMPVSTFDDIAPY      160
LC_01016        IAAVLIFIFLFYTTCVRVIDPVVSSIVSGSPADIAGIKPKDRLLSIDAVNISNVGDVYSY      97
LAm_00185       ITAILIFIFIVYNSPMPMIDPVVSDVISGSPADIAGIKSKDRLLSIDGLKISNIRDVHSY      163
              : : ::  *:.*..   ::. :: .:   :**    *:*  * ::*:*    :*    ::  *

LCres_00080     VQSHPKKEMIFILKRQDKDFVKFAITPRMEKITNALGQKVSVPLIGLKVYSVKSKHQFLT      239
LAf_00496       IRKNQLREVEFVLQREHVGIITLKVTPRLQDFIDQFNVKHKIPTIGILF--DSGNLHYRT      217
LAs_00429       VRENPLHEISLVLYREHVGVLHLKVMPRLQDTVDRFGIKRQVPSVGILFSYDETKLHSRT      219
LPsy_00233      IRENVSKEIVVGVHREYVGILKLKVVPSFLDFVDRFGVKRRIPSIGISFNYDKTRLQYRT      220
LC_01016        LNSNITKEMKVILSRDHVGTLTVKVVPQTRYVVNNFGIKKKIASIGVSFNNSTVRLQYRS      157
LAm_00185       LNTNLSKEIKIILYRSNVGEITVKIIPQTKYIVNNFDIKNSVLSIGVNFDSSVIHYQYRS      223
              :. :  :*: . : *.  . : . : *      : :. *  :  :*: .      . :  :

LCres_00080     LPEAFFMGLYEIHDIVKATLQYFYNVLSGQMKSDQITGPIGVAKIAKHMSDIGFEALVRF      299
LAf_00496       VLQSFSRSLNEVISITIKSFSGLIHIFSGDVKVHSIHGPVGIAKVAKKFAEYGFNSYIEF      277
LAs_00429       VLQSFSRGLDEISSITRGFLGVLSSAFGKDTRLNQISGPVGIARIAKNFFDHGFNAYIAF      279
LPsy_00233      VSQSFLRGLKEMGLITQRTLSVLSNIFSRDIK-YQISGPIGIAKAAKDFSDQGFDSYIGF      279
LC_01016        ISESFLNGLNYSFYIIQRTIVSLYNILTGSIKADQMAGPIIIAKLAKEFASEGFNAYIEF      217
LAm_00185       ISESILKGLNYSFDLMRSNVIALRDILHGKVKSSQIIGPIAIIKIAKNVASEGFKSYIDF      283
              : :::  .*     :     .  :   :  . :  .: **: : : **.. . **.: : *

LCres_00080     LAFISLSVGLINLMPIPILDGGHLMLYFLEAVRGKPLGELSEKIVLQIGLILVLVLTVVA      359
LAf_00496       LAIFSWVTGFMNLLPIPILDGGNFMIFLFEMIRGKPLKVSTVRFITKIGCSFILFLLFLG      337
LAs_00429       LAMFSWAIGFMNLLPIPILDGGHLITFLLEMIRGKSLGVSVTRVITRMGLCIILFLFFLG      339
LPsy_00233      ISFFSWMAGFMNLLPIPILDGGNVVIFILEMIRRKPLEVAVARVITGIGICIILVLFMLG      339
LC_01016        VAILSISIGLFNLLPIPLLDGGNFVVFLLEIIRGKPIGKSAETIIMMIGLLVVITLSVLI 277
LAm_00185       VAVLSLSVCFFNLLPIPLLDGWNCWVFLLEMIRGKSIGKLAEMIIMIIGLFIVITSFVLI      343
              :::.:*    ::**:***:*** :   :::* :* * :      .:  :*   .::    .:

LCres_00080     TRNDVSGLINSFKQ---------      373
LAf_00496       ISNDIYGLLW-------------      347
LAs_00429       IRNDIYGLMQ-------------      349
LPsy_00233      IRNDIYGLIK-------------      349
LC_01016        ISNDIYRLIGWFLEMNNGFIDVL 300
LAm_00185       IGNDIYRLIS-------------      353
                 **:  *:
```

CLUSTAL O(1.2.4) multiple sequence alignment

M23 family metallopeptidase

```
LCres_01145     ----------------------MCTNNLCFFKKKLNEFL-------LSITIVGIIS----    27
LC_01051        ------------------------MNLVCKCINRLYSSHAIYFIKM---LVFTIISIFSN33
LAm_00308       ------------------------------------------------------------    0
LPsy_00451      ------------------------MNVFYRYINKYYKIL--------AKTIFCFLLILVS    28
LAf_00312       MRSTTVFLIKYKMIIYSYIRSIKQVNKSFLSIHRLFVYPPHYKIYKILINISAWIMIVMP60
LAs_00521       MRSTILFLIKHKLKLHKNIMSKGKINESFWYIRTLFPYLSHQIHKALMSISSGIILILSP      60


LCres_01145     -GCSTTKTDPIFISPTNNQNPPENNTKYLPVPDIDVGTNSLEPLQATENTINNNEP-LSN      85
```

```
LC_01051        QNATAES---AK-------YYNAQINKYHPKPQFLF------------------------ 59
LAm_00308        ------------------------------------------------------------    0
LPsy_00451      TSDKTNAE-NIL-------VLPSKITNYHNRLVEQKNNPEKQV-------I--PIPLPTK         71
LAf_00312       VDCKADIK-----------TPTDSINDQ--QIVILKKNKKLRHIP-----I--PDLSATK             100
LAs_00521       TDCTANTA-NIL-------IPSRKIIDYH-RLLEQKKNHNLQYSL-----I--NIPSQNK            104


LCres_01145     QTYPVYQT----EPIERVERTSLFQKKATHSNLSVNKPAAAASKNAHISNHTNSFKKNKK                141
LC_01051        -------------------------------ELYKNR-----------------------                65
LAm_00308        ------------------------------------------------------------    0
LPsy_00451      QEYPSNTNNKTIDQNTLLAKKGFVDKNNLYSSMYLSKKIP-------LPNK---------      115
LAf_00312       KGSPKSDPNKTSDHIDRLKEKLLADKNNSYDN-HLNTQIP-------LPNN---------      143
LAs_00521       QESPKNANNNILDHIALLKERLRTDINTFDNT-NLETKIP-------LPNN---------         147


LCres_01145     KTIFLSEKKSSSLSSSTTNSYKTENKPVNSKQKERYNNKSVENKNIYTVQVGDTLEKISQ              201
LC_01051        -ITPPPPKKPISQ-----NNRLSNNNRLKRK------------NKISTARNTRQT----K             103
LAm_00308        ------------------------------------------------------------    0
LPsy_00451      CLLFPPDNNSLHL-----NNCIENKSPNSSK------------KNISHTRKIPKY----K            154
LAf_00312       ----------LQA-----NSQIEEKFKQ----------------NISAIKKDQ-------        165
LAs_00521       ----------LKP-----NVCVKEKKLI-----------------------PP-------            162


LCres_01145     KMNINVNSLKVRNNITGDAIRPGQTLVITQENSSGKAIDSSITESKGKPLKNVVSKNNIN               261
LC_01051        KNNPN--LN-------K-------------------NIYPSFTIPRTQTIKNTNDLDDKT                  135
LAm_00308        ----------------------------------------MIPRARTIKSTNDLDNKN          18
LPsy_00451      KNNPK--KS-------G-------------------NIAPAFKVKNNQKIKHIQYKKNNY                 186
LAf_00312       KKNIN--NL-------G-------------------NTDLKTKKNQNKHIH---------          188
LAs_00521       RKNIN--NL-------K-------------------DTNHRLKIKNNQEIKNIHHKKNNH                   194
                                              . : ::

LCres_01145     L-SKNYVKNSTVPNTQQKPTLNDKIPKLTGIGKYRWPVTGTIVNSFGKNSAGQYNDGIDI              320
LC_01051        CPKSISGENNPTNNDPIGKTSNTISYSDKKSEKYIWPVMSNKITFS------PNNNGIDI                   189
LAm_00308        NPKIISDKNHVINSYSIEKTPNDISDKDQQFTKYIWPIMSNKFTFS------RNKNGIDI                 72
LPsy_00451      SPQIHPIKNQKTKNNFPKSTVKTIENNQNKSEGYLWPVKGNIVNFV------KNNNGIDV               240
LAf_00312       ------------------NIYSISDNKQKKPSKYFWPVTGDTINFT------KNNDGIDI              224
LAs_00521       AYTVS------NKNTSNYLFPKTIENNQRKLRKYFWPVTGNIVNFK------KNNNGVDI 242
                           .    .      * **: .  ..          ::*:*:

LCres_01145     SVPSGTAIKAAENGVVIYVGNSLKKLGNTVLIRHADGIVTVYGHAESISVQRGQKVQRGQ             380
LC_01051        FIPPDTAIRSAKDGIVIYAGNDLVDLGNTIIISHNNSISTVYGHASAIYVKKGQKVTSGQ                 249
LAm_00308        FTPPDTQIKSAKNGIVIYAGSDIRELGNTVVICHNNSMSTVYGHLNAIYVKKGQKVIRGQ             132
LPsy_00451      LVPPNTPIRAAKDGIVIYVGNDLIELGDMILIRHDNEMVTVYSHINTPYVQKGQKVSRGH              300
LAf_00312       VVPPHTPIKAAADGMVIYVGDDLVELGNIILIRHDSSTVTVYSHIDKIYVHKGKKVSRGY            284
LAs_00521       LTPPNTSIKAAADGMVIYVGNDLVELGNTILIRHDDSIVTVYSHIDTPYVQKGQKVSRGH             302
              *  * *::* :*:***.*..: .**: ::* * .   ***.* .   *::*:**  *

LCres_01145     TIAISGMSGDAKRPQTHFEIRKNAIALNPLNFLE----------            414
LC_01051        IIALSDNISNTKKSKLYFELRKDAVALDPIGYLEHKHNLKDNKK     293
LAm_00308        TIALSGNSKNTKKPKLHFELRKDIVAIDPLEYLDPKHNLK----        172
LPsy_00451      TIGISRISDDKKISKVHFELRQNAIAVDPIAFLEKTSYTQKSDQ     344
LAf_00312       TIGRSGTSGKTKQSTIHFEFRKNTIAMDPIKFLEEGDST-----         323
LAs_00521       TIGLSGKSGNAQHPQVHFELRKNAIAMDPIKFLEEKIP------         340
              *. *    . :    :**:*:: :*::*: :*:
```

CLUSTAL O(1.2.4) multiple sequence alignment

disulfide bond formation protein B

```
LCres_00252     MNRILSLTLTKRQLTILLMIGMSTVLILTLVLEYVDEYIPCELCLKERNAYYYCLPISIL                60
LC_01063        MFISAISKLTSLHVIRILIIGFAFVLIYSLALQYIAGYEPCKLCIQERSIHFYCLLITIL    60
LAm_00281        MFMSSISKLTNLHIIRILIVGFTVVLICSLALQHIAGYIPCKLCLQERSLHFYCLLIAII60
LPsy_00417      MIKSLLSKLENIYIIRIITTIIAGIIISFLTIQHVGGYAPCDFCLREQRPYYYCFLMAIA 60
LAf_00345       MIKSLLSTSNNVPLIRIILISITGIIICFLTIQYIGGYPPCHLCMQEQRIYYCGFIIAIT    60
LAs_00240       MIKSLLSTLANIPLPRIILLNISGVIVCFLMIQHVGGYPPCDLCIQEQKIYYFGFLIALV                60
              *      .  .  :  ::   :: :::  * ::::  * **.:*::*:  ::  : :::

LCres_00252     AAILGRNEKFSKLISFLMITISFIMLSNAIIGSFHTGLERGLWSGSKICTLEDTNSNNLT               120
LC_01063        ASFTSYNNRLYWITCQLLIAVSMIMIYNMVLGIIHFEIELNILPAGNACIIKSI-DEGI-   118
LAm_00281        ASFTAYNNRLYLITCILMGLVSIIMIYNIVLGIIHVEIELNILSAANACIIKPM-NEEI-    118
LPsy_00417      ANFSIQNNRLYRTTFFLLMTISLVMLYDTIISIIHVGIEWNIWRENTICTNDSK-IESVK               119
LAf_00345       INLSCQNRRYHNINFYLMLMLSLLMILNIIISVIHIGIEWGIWKNSAICTDKSK-IESIT 119
LAs_00240       ADLSTRNHNSYWSTRLLLMTLGLLMFFNMTISVIHVGIECGIWEKNAICMNNSK-IESIT              119
               :   *..       *:  :.::*: :  :. :*  :* .:       *   .     : :

LCres_00252     NAKDLFSKIKTSNKKSCNEVQLYILGLSLASWNVITSFFFAIIAFQASRKTLKENF----               176
LC_01063        SAADLSASIGQQSIISCNKVSLHVLGLSPASWNIVLSAMFSIISYIAAIKTFRNQSSKRN               178
LAm_00281        SAEDLSSSIENQAIVPCNKVNLNILGLSPATWNIIYSVIFSIISYVAAIKTFRKSYPHK-              177
LPsy_00417      NTIDLLTHMEREHILRCNQTKLYILGLSLAFWNVILSFMLSVLSYIAAGKTSSFSDNNFK            179
LAf_00345       STKDLLTQIGKDLIHSCNKSKLYILNLSLVWWNLILSFSLLYMTSIATYNLFKKTNEH--              177
LAs_00240       STVDLLTQMEQENIPSCNKTTLYVLGLSLAFWNIIVSFFLSFITSIAMLKISRKK-----174
              .: ** : :  .     **:  * :*.** . **:: *  :  ::  *  :

LCres_00252     ------------         176
LC_01063        ANNPNHYQNDTR                     190
LAm_00281        ------------         177
LPsy_00417      K-----------         180
LAf_00345       ------------          177
LAs_00240       ------------          174
```

CLUSTAL O(1.2.4) multiple sequence alignment

peptidylprolyl isomerase

```
LCres_00277     MKEILDHVGVGEKHFKNFLSIQMAWPYVVSVHSEQ-----NTD-TPITGNKKPNPTTTEY                171
LC_01111        MINALETQGIGENHFKEYLATQHFWNEFINSSFNFKNSGFKIESSPNRNT-RENITIKEY                 59
LAm_00297        LINKLETQGIGEKHFKEYIAVQIYWNEFINRNFRFNNSAIKQTNPNNLKI-NNDIKIKEY                174
LPsy_00436      FSDMLEHLGIGENHFKQYLAIQLAWDEIVRNTFIMKYRGLGMEMPPSIKGRGKNLTVREY               175
LAf_00326       FISLLNEKGVGENHFKKYLAIQLTWGDFIKRDFMFKHQNIEREMPPNIQKMKNNTRVHEY              178
LAs_00269       FSSFLDKQGIGDNHFKQYLAIQSIWPDVVKNDFMLKYGNLEMEIPANKQKMKN-ITVREY              178
              : . *:  *:*::***:::: *  *  .:                              **

LCres_00277     ILQQIIFIIPQSFSASKRNVMIKKRKKEAEASRSKFP-GCEKAKEFASKMRDVYVSDSQR              230
LC_01111        TLKTVTFITPEKNS--NNADYIKQRIKEAEDSRAKFPKDCSKAEEFASKMMDVSIGEEQR              117
LAm_00297        ILKEVTFVIPNKKS--DNTDYIKQKIKEAEASRAKFPKDCSKAEEFASKMVDVSVGEGKR             232
LPsy_00436      LVKKIIFSVPYNKH--KNEDFIQKRIDEAEKSRFHFPTNCNRVEEFASAMHDVSVSNPQY             233
LAf_00326       LIRIMIFSIPDDKI--KNEDFINKRMDEAQESRFKFPKDCNNIENFASKIHDVSVSKPQY             236
LAs_00269       LIRTVLFSIPDNKL--QNQGFVQKRIKDAEESRLRLPKDCNKLEKFASKIHDVSIGKAQY              236
               :: : *  * .    ..   :::: .:*: ** ::* .*.. ::*** : ** :.. :

LCres_00277     LLEPMLSPEFKKLLNKIKNGTTPTHVTEKGIEYISICSQRQVNDDTASLVISKATDFNKK               290
LC_01111        VLETNLHPRIHNLVTKARSNTTDVYVTQAGVEYIAICDKIDIGGEFALNEKFKYQEIPKK               177
```

```
LAm_00297     ILETNLNPKVQDLIKKASNNTTDVYMTQSGVEYIAICNELDIGGEFALNEKINYQEISKK     292
LPsy_00436    FLESDLQPQLKILLKKTKNNTTNTYVTEKGVEYIAICNIRDIGGELALKAYFNTQEIPKK     293
LAf_00326     IVEYDLNPRLQELLKKEENNTTDPYVTERGVEYIAICSKKDLNGEIALQEYFKNHNIPTK     296
LAs_00269     LLESDLHPQFQNLLKKSQNNTTNPYVTQKGVEYIAICDKRDLGGEIALKAYLSAQNTPTK     296
              .:*  * *..: *:.*  ..**  ::*: *:***:**.  ::..: *     .  :  .*

LCres_00277   QQKNSEQYIADLRSKARIRYY-             311
LC_01111      IEQYEKEYIKAIRKSAHIRFYK   199
LAm_00297     IEKYEEEYIKNIRKNAHIRFYK   314
LPsy_00436    MKKYDEEYMKTLRANAIIQFYQ             315
LAf_00326     MEKHSEEYMEKLRSNANIHYY-317
LAs_00269     IEKHEAEYVKKLRSNAIIHYY-   317
               :: . :*:  :* .* *::*
```

CLUSTAL O(1.2.4) multiple sequence alignment

4-hydroxybenzoate octaprenyltransferase

```
LCres_00849   PLYPFTKRFTNWPQFFLGLCFAWGALIGWSALHNKLSLAALCLYAGTVCWIIGYDTIYAH     210
LC_00039      ---------MSCPQVILSLCFSGGIIIGWAAIYGSISLATILLYIGSICWVIGYDTIYAH     51
LAm_00431     VLYPFSKRFMKCPQIVLSLCFSGGIIIGWSSIYDSISFVTIFLYIGVMFWVIGYDTIYAY     205
LPsy_00856    LIYPFSKRFIPCPQVVLGICCGGGVFIGWGSLHESFSWTAFWLFIGTIFWVIHFDTVYAH     239
LAf_00084     LLYPFSKRFIPCPQVVLGIGFAGGVLFGWGALHASLSWPAFWLFIATIFWVVYFDTVYAH     205
LAs_00080     LLYPFSKRFILCPQVVLGIGFAGGVFVGWGALHASFSWPAFFLCIGTIFWVVYFDTVYAH     207
                **..*.:  . * :.**.::: .:*  :: *   . : *:: :**:**:

LCres_00849   QDKSFDELIGVKSTAILFADHTKRWLCLFYSLCIVFLALSFYLVEVTIYSWIGLLSAALL     270
LC_00039      QDIEDDELIGLGSTARVFANNTKAWLLFLYGMFIFLFITAFHIIHVNIFAWIGLLIVLFL     111
LAm_00431     QDIEDDELIGLGSTARVFANNSKLWLFFLYGLFIFSLVVSFYLINVNIFAWIALLIVFCG     265
LPsy_00856    QDKKDDEQIGINSTARLFADHTKIWISILYGLFTLCFMIALYLVQANFFAWIGLLIALFF     299
LAf_00084     QDKKYDEKIGINSTARLFADQTKIWLFVLYGIFILCFAVAFYLVQVNFVAWIGLFIALLL     265
LAs_00080     QDKKDDELIGVNSTARLFSYQTKLWLFILYGIFVLCFMVAFCLLTVNFFAWIGLLIALFL     267
              ** . ** **: *** :*: ::* *: .:*.:  . :  :: :: ..: :** *: .

LCres_00849   FIYQIIAFDGSDPDKCLALFKSNNFTGLLVFTGLLISLLSI--        311
LC_00039      LIKQIVVLDTSCPDKCLIAFKENDTCGMLLCLGLIMSLIVF--        152
LAm_00431     LMKQIITLDISCPDSCLISFKDNDIYGMWLCFGLIISLITC--        306
LPsy_00856    TIKRIIILDISSSDQCFLFFKTADMTGMFIFASLLFSLFLDQF        342
LAf_00084     TIKRIITLDFSCPRQCDMFFKHADYVGIFIFVCLMISLFLDQL        308
LAs_00080     TIKRIIALDISCSKQCCSFFHSAGVVGILILICLIISLFLDQF        310
               : :*: :* *   .*   *:  .  *: :   *::**:
```

CLUSTAL O(1.2.4) multiple sequence alignment

DNA polymerase III subunit chi

```
LCres_00282   GIDVGEEASFASRQPVLLTVFCDNPNNAHVRILIDNAFVNIETVLSYESVIIMFDDYDKN     120
LPsy_00441    GIDVGEEEALASFQPILLTVSSNNANASMVRFFVDQASMHIGDMDHYKKIIFIIDTDDQA     119
LAf_00321     GVDVGDEEVFSSFQPVLLTISSLNANSSSIRFFVDRASFQMRDVDLYKKLVFIINTDDQE     119
LAs_00264     GVDVGDEGDFSSFQPVLLTISSLNANTSTIRFLVDEASMHIGDVDYYEKVVFIINTDDQG     119
LC_00364      -------------------------------------MHNDDIGRYDKIVFIVDNNDQE     22
LAm_00302     GIDTGEESELSTFQPILLTVNGDNANSSTIRFFVDKALMCNDDIGNYEKIVFIVDYNDKE     119
                                        .    :  *..::::.:  *:

LCres_00282   QLETARSEWKNLKAQGYKLSHFQKNKTGLWEKKGIK          156
LPsy_00441    SLKWGRANWKNLKSNGYKLTFL--------------     141
LAf_00321     SLEWGRENWRNLKNRGYSLDAL--------------     141
LAs_00264     SLEWGRAHWRSLKNSGYAPDVI--------------     141
LC_00364      SREWGLKNWHRLKNDGYKLIVH-HNNSGLWQTLYF-           56
LAm_00302     NREWALKNLLSLKNNGYEPTIH-HNASGTWKKL---           151
              . : .  .   **  **
```

CLUSTAL O(1.2.4) multiple sequence alignment

DUF1217 domain-containing protein; similar to FlgF

```
LCres_00851   EELSKKEFHSLLASDILDPHSDVNRSRDQRFIQLNRAFNFDSNGNASSPFQALSEKTIQD     470
LC_00921      ------MLRDVLTSDPSDPNSYINKYKDKRFKDLNLAFNFDKNGKAKQMPIAHLPLTKRD     54
LAm_00746     -DTYKYVLRDVLTSDPNDPNSYINKHKDKRFINLNLDFNFDKNGNPKKMPMAHLPLTKKD     464
LPsy_00721    NDLNKYRLRDILISDSSDSKSYVNQLKDNRFINLHHAFNFNKKGEIGFNRLTQSKLTLRD     467
LAf_01016     EDLDKYKLKSVLTSNASDPNSYVNKSKDKRLIKLNNAFNFNSNGSIGEIPTIQSRSVIRE     463
LAs_00907     QDLDRDQLRNVLTSNPSDPDSYANKSKDDRLIKLNHAFNFNADGSIGPIPMMQSRLTIRD     464
                ::.:* *:  *  .*   *: :*.*: .*:  ***: .*.          . ::

LCres_00851   YRDSYIKNKTRFLTQLE------------RKKNEKDLNPEITYFEENIRKIHHPDDFTAD   518
LC_00921      YIQDYIKNKMHDYINSYETIFAKKGQ----RKVEENIRSNIDYYMENIDKIDSFENLIAD     110
LAm_00746     YIAGYVKNKNNDYINNDKVIFSKKGQ----IKIGENIRSNVDYYINNIDKIDSFENLIAD     520
LPsy_00721    NVMNYIGYKKSLYNE--NKIFSSAQNIR----IKKDTDKEVEYYTSNIQTIHSFNDLLAN     521
LAf_01016     NIINYVNSKRNLYNE--KKSFDSENKIHM----EKKLDSEAKYYSENIDSIHSFEDLFTN     517
LAs_00907     NVKHYANRKRSIHDA-ENKSFNSMEQISTQKRINLEINSEVDYYTLNINTIHSFEELLAN     523
                *   *                     .  :  *:  ** .*.  ::: ::

LCres_00851   KRLVHFMLEAKGIKPDTVTENLLKETFTSYFNTPDNFIHTSNDYRFMEIVASFHFDANKK     578
LC_00921      KRLMDIILESKGIDSKKVSKVFLQYIFQSDLKDPKSFANKYKDQRYKEIICSFDFDLINK     170
LAm_00746     KDLMHIILDSKGIDSKKVSKSFLERIFKSDLADPKSFVNQYKDKRYKEIMLSFEFNSINK     580
LPsy_00721    KRILNFLLESKEIDPKKVKNSFLEKIFKSDLNNTKSLANTYKDSRYKEIMLSFNFDIHGT     581
LAf_01016     RKILNFLLESKEIDPKKVDDNFLHKIFRSDLKNPNSLANTHQDSRYKDIISSFNFDIRGE     577
LAs_00907     KRILNFLLESKGIDSQKVREDFLCEIFKSDLKDPKSFANTYKDNRYKEIISSFNFDIHGE     583
              : :..:*::* *. ..* . :*   * * :    ..: :  :* *: :*: ** *:

LCres_00851   LSRNSAHQIQSPENIMKTMHLHTHQILEEEEGKKDINRRLALYFERKAPTITNIYDFLSD     638
LC_00921      KIDKNHGKIQNDFHINKTIESYKHQILESEVGQRNLDMELALYFQRVIPNISSIYEILAD     230
LAm_00746     KIYKNNGKIQNYFHINKTIESYKNQILEDEVKDKNPDMELALYFSRIIPTINSTYEILSD     640
LPsy_00721    KSNENTGKVQDNFHIDHVVDLYKNQILQKKEEEKDPDVALLLYFKRTIPTIKNIYDILGD     641
LAf_01016     KLYKEKGAVQDDLHLDKTCDLYKNQIIEKEEEQRDPDNALEIYFKRNIPNIRNYYEILGD     637
LAs_00907     LSHKKIGKVQDNFHIDDTCDLYKHQMIEKKEEEKDPNIALALYFKRTIPNIRNHYEILGD     643
                :.   :*.  :: .. . ..:*:::.:  .:: :  * :** *  *.*  . *::* *

LCres_00851   KALYQVISKTLGLSSYFRSGDIDKQAATLKKSINIKDFQDPEKLKNFIKRFNSATNSENS     698
LC_00921      KNLFKVVSKVCKLPIHFSSLHQKKQIDTLKKHIDLKDFQNPKKVQQFLYKFGASNIEKSA     290
LAm_00746     KNIFNVVSKVCNLPVHFSSLNQKKQIDILTKKINIKDFQDPKKVQQFLYRFFYTDNVKRT     700
LPsy_00721    KNLFQVISKKLRLSPHFPSLPESTKIHILKKLINIKDFKDPKKLENFLYAVNVNHYQSTT     701
LAf_01016     KKLFQVVAKKLNLSPYL---PEKNKLDMLQKNINIKDFKNPKKVDAFIYAFRANKFKNID     694
LAs_00907     SKLFKVVSKKLKLSPYFAVLPERKKIRILKKHIKINDFKDSKKVEDFLYAAKAMDHQSID     703
              . :::*::*   *  ::       .:    * * * .::**:: :*:.  *:        .

LCres_00851   VLIPGFNSSIK-----------------------------NSGIFSLIQSLPLS------     723
LC_00921      QHVSLFYQT--NTQDNLYDNSFPE---I-------LPLSLNNNKLSFIVDRSNKPESYNY  338
LAm_00746     DQSVLLLSQ--SDQYDLYNEKLPE---V-------STLSLSGTKLSLLDLFNKSSAQINY 748
LPsy_00721    HKIALTLAFFPPL----EKDSMDDHEKNISLQL--SPNKKENGKFSILNLFS--------     747
LAf_01016     YKKSLF--SFANKQERYLYDYPENQENTMSLQLNTINPRPSNGRYSTLDLFIPLN-----     747
LAs_00907     YKKALF--SSPNQNKDPWNNSFDDPQNNVSLQL--SPQKKSNGRFSILNLF---------     750
```

```
                                  ..   * :

LCres_00851     ------------------- 723
LC_00921       FTDIATDYLYTLLSGSQMI        357
LAm_00746       FKGFFSR------------       755
LPsy_00721      ------------------- 747
LAf_01016      -----SPSFLSL-------        754
LAs_00907       ------------------- 750
```
